# Microbiota Analysis of Chickens Raised Under Stressed Conditions

**DOI:** 10.3389/fvets.2020.482637

**Published:** 2020-10-07

**Authors:** Rabindra K. Mandal, Tieshan Jiang, Robert F. Wideman, Troy Lohrmann, Young Min Kwon

**Affiliations:** ^1^Department of Poultry Science, University of Arkansas, Fayetteville, AR, United States; ^2^Quality Technology International, Inc., Elgin, IL, United States; ^3^Cell and Molecular Biology Program, University of Arkansas, Fayetteville, AR, United States

**Keywords:** chickens, stress, bacterial chondronecrosis with osteomyelitis (BCO), gut translocation, extraintestinal microbiota, *in ovo* colonization

## Abstract

A substantial progress has been made toward understanding stress-associated gut and extraintestinal microbiota. However, a comprehensive understanding of the extraintestinal microbiota of chickens raised under stressed conditions is lacking. In this study, chickens were raised on a wire-floor model to induce stress, and the microbiota in the gut (ceca) and extraintestinal sites (blood, femur, and tibia) were characterized at different ages (1, 17, and 56 days) using 16S rRNA gene microbiota profiling. Open reference OTU picking showed extraintestinal sites had a significantly higher number of unassigned OTUs compared to ceca across all ages of chickens. Extraintestinal sites of all ages, irrespective of body sites, as well as ceca of 1 day-old chickens had significantly lower alpha diversity than ceca of older chickens. Intriguingly, bacterial diversity (alpha and beta) and OTU interaction network analysis showed relatively stable bacterial composition within the extraintestinal sites of chickens regardless of age and sites compared to ceca. Furthermore, assessment using UniFrac distance suggested the gut as a possible source of extraintestinal bacteria. Lastly, LEfSe analysis showed that both commensal and pathogenic bacteria were translocated into the extraintestinal tissues and organs under the stress. Extraintestinal sites have highly abundant novel taxa that need to be further explored. *In ovo* microbiota colonization and/or translocation of circulating maternal blood microbiota into ovarian follicles might be the source of intestinal and extraintestinal microbiota in 1 day-old chickens. Our comprehensive microbiota data including extraintestinal sites in reference to gut provide unique insights into microbiota of chickens raised under stressed conditions, which may be relevant in other animal species as well.

## Introduction

Stress is a condition where homeostasis of an organism is disturbed (1). Stress increases intestinal permeability, and pro-inflammatory activities including other effects that aid in the translocation of bacteria from the intestinal lumen into blood circulation. Stress activates intestinal mast cells to secrete pro-inflammatory cytokines such as IFN-γ and TNF-α. These pro-inflammatory mediators increase epithelial and endothelial paracellular permeability via the disruption of the cytoskeleton and reorganizing of the structure of the tight junction (2, 3).

Subsequent research has shown that gut microbiota in humans can translocate into extraintestinal sites under stress or disease status as in the cases of pancreatic lymph nodes in Type 1 diabetes (4); blood and liver in liver fibrosis and cirrhosis (5, 6); mesenteric lymph nodes, liver, lung, spleen, and blood in acute ileitis (7); blood in multiple sclerosis (8); peripheral tissues in stroke (9); portal and systemic circulation in human immunodeficiency virus (HIV) infections (10); blood in inflammatory bowel disease (IBD) (11); and tissues in major depressive disorder (12). In mice, the composition of cecal microbiota was correlated with stress induced by the grid floor (13). In chickens, oxidative stress, poorly digestible protein, and coccidiosis are mainly responsible for a leaky gut that leads to the translocation of gut microbiota (14, 15). Previously, we have shown that blood, tibia, and femur microbiota are associated with bacterial chondronecrosis with osteomyelitis (BCO), a disease caused by stress on the leg bones of chickens (16, 17). Despite numerous studies on translocation of gut microbiota due to stress, a comprehensive assessment of extraintestinal microbiota of chickens raised under stressed conditions is still lacking.

In this study, a wire-flooring model was used to induce stress in broiler chickens to increase the incidence of lameness through BCO (18). Chickens raised on a wire floor are stressed due to unstable footing accompanied by the rapid growth rate; this causes microfracturing in cleft formation in the proximal head of the femur and tibiotarsus. Bacteria from the gut spread hematogenously via intestinal translocation to the fractured cartilage of the femur and tibia and promote generalized necrosis, causing BCO (16–18). Covering the entire surface of a pen floor with wire flooring also subjects broilers to behavioral stress due to their inaccessibility to litter. In this study, chickens were raised on a wire floor and ceca and extraintestinal (blood, femur, and tibia head) microbiota were characterized at 1, 17, and 56 days of age. Interestingly, the results indicated that 1 day-old chickens harbored extraintestinal microbiota which remained relatively stable throughout 56 days in all sites except for the tibia. Our comparative analysis of beta diversity distance suggests that *in ovo* microbiota colonization and/or translocation of circulating maternal blood microbiota to ovarian follicles might be the source of intestinal and extraintestinal microbiota in 1 day-old chickens.

## Materials and Methods

### Animal Experiments and Sample Collection

Experimental design, animal housing, and management are described in detail in a previously published study (19). Animal procedures were approved by the University of Arkansas Institutional Animal Care and Use Committee with approval number 11002. Briefly, all of the chickens were raised on wire flooring from 1 to 56 days of age. Cobb 500 chicks were initially placed at ≥60 per pen. Chickens were given *ad libitum* feed and water. They were culled to 50 per pen on day 14, yielding an experimental density of 1 ft^2^/chick. Samples were collected from the ceca (1 ceca per bird) and three extraintestinal sites: blood (5 ml per bird) and 2 leg bone sites (1 femur head and 1 tibia head per bird). All samples were taken from clinically healthy chickens at 1 and 17 days of age (hereafter referred to as day 1 and day 17), and from clinically lame chickens exhibiting BCO symptoms and lesions at 56 days of age (hereafter referred to as day 56). Four samples per bird were collected from 16 chickens (*n* = 8 on day 1, *n* = 4 on day 17, and *n* = 4 on day 56) totaling 64 samples. The birds in all pens were “walked” and observed for lameness every 2 days beginning on day 15. Birds that were unwilling or unable to walk were diagnosed as being “clinically lame” and were humanely euthanized by CO_2_ gas inhalation.

The selected birds for sampling were humanely euthanized by CO_2_ inhalation and transported to the lab within 30 min post-mortem. All samples were collected in a laminar flow hood. For leg bone samples, femora and tibiae of both legs were exposed for scoring based on macroscopic lesion appearance and sampling. Forceps and shears that were dipped in 95% ethanol and flame-sterilized were used to aseptically remove the upper metaphysis and physis (growth plate) of each proximal bone end. Forceps and shears were re-sterilized immediately before collecting each bone sample. The cut surfaces of the proximal femoral and tibial samples were dipped in 95% ethanol, flame-sterilized, dropped into sterile culture tubes, and stored at −20°C for subsequent DNA isolation.

### DNA Isolation, PCR Amplification, and Amplicon Purification

For cecal samples, the contents were squeezed out of ceca into sterile Nasco whirl-pak sample bags and 10 times volume (v/w) of sterile phosphate buffered saline (1 ×, hereafter referred as PBS) buffer was added to each sample. The cecal contents were resuspended in PBS buffer by gentle mixing with hands, and 200 μl of each sample was transferred into a microcentrifuge tube for DNA isolation using a QIAamp DNA Stool Mini kit (Qiagen, Valencia, CA., USA). For blood samples, 1 ml of the whole blood collected in Vacutainer (Becton, Dickinson and Company, Franklin Lakes, NJ) was transferred to a microcentrifuge tube and centrifuged at 5,000 rpm for 5 min. Two hundred microliters of buffy coat were collected from each sample and used for DNA isolation using a QIAamp DNA Mini kit (Qiagen, Valencia, CA., USA). For bone samples, PBS buffer of 10 times volume (v/w) was added to each sample, and the samples were homogenized using Bullet Blender 50-DX (Next Advance, Inc., Averill Park, NY) for 10 min following the manufacture's instruction. Two hundred microliters of the homogenized sample were transferred to a microcentrifuge tube and used for DNA isolation using a QIAamp DNA Mini kit. The concentrations of the purified DNA were measured using Qubit 2.0 fluorometer (Life Technologies).

PCR amplifications were carried out for each sample to amplify V1–V3 regions using 27F and 533R primers with Illumina adapter sequences and unique barcode sequence attached at the 5′ end of each primer. The PCR conditions consisted of approximately 100 ng of purified genomic DNA, 10 × Buffer II, 5 U AccuPrime Taq DNA polymerase (Thermo Fisher Scientific Inc, Waltham, MA USA), 1.2 μM each primer in a total volume of 50 μl. The DNA engine thermal cycler (Bio-Rad, Hercules, CA, USA) was used with the following amplification cycles: 94°C for 5 min; 30 cycles of 94°C s for 30 s, 55°C for 20 s, 68°C for 30 s, and 68°C for 5 min for final extension. Each PCR product of the correct size was gel-purified (Qiagen, Valencia, CA, USA). PCR products of all 64 samples were mixed in equal quantities, based on measured concentrations. Illumina sequencing was performed for 300 cycles in a paired-end mode (2 × 300 bp) with Illumina MiSeq at the Genomics Core Facility at the University of California, Riverside.

### Data Analysis

The paired end reads were joined using Quantitative Insights Into Microbial Ecology (QIIME 1.9.1) with join_paired_ends.py scripts (20). The barcode sequence in the forward and reverse reads were brought together using custom Perl script discarding only the 8 nucleotides random sequence from forward read. The barcodes were extracted with extract_barcodes.py and assembled reads were mapped to the respective sample with split_libraries.py. Chimeric sequences and reads lower than 400 bp and >600 bp were discarded before operational taxonomic unit (OTU) picking. OTU table was created using QIIME's open-reference OTU picking method with a default clustering algorithm (uclust) against Greengenes database (gg_13_8_otus) at 97% sequence similarity (21). The mean number of sequences per sample was 43,739.28 (SE ± 3,824.32) with a minimum of 2,615 and a maximum of 147,499 sequences (Supplementary Table 1). Taxonomic assignment, alpha diversity, linear discriminant analysis (LDA) effect size (LEfSe), and bacterial networks were performed on normalized OTU table using cumulative sum scaling (CSS) (22). Beta diversity between the different groups of chickens was measured with unweighted and weighted UniFrac distance at an even sampling depth of 600 sequences/sample.

Additionally, unassigned OTUs were aligned against the reference genome sequence of *Galus gallus* (GCF_000002315.6_GRCg6a) using the Burrows-Wheeler Aligner (0.7.17-r1188) with a bwa mem algorithm (23). The OTUs that matched to the host genome with >95% coverage and >94% identity only were considered to have a significant match.

Statistical analyses were done with GraphPad software (La Jolla, CA). The bacterial network was drawn with Cytoscape (24).

## Results

### Bacterial Composition in Body Sites of Chickens Across Different Ages at Phylum Level

**Figure 1:**
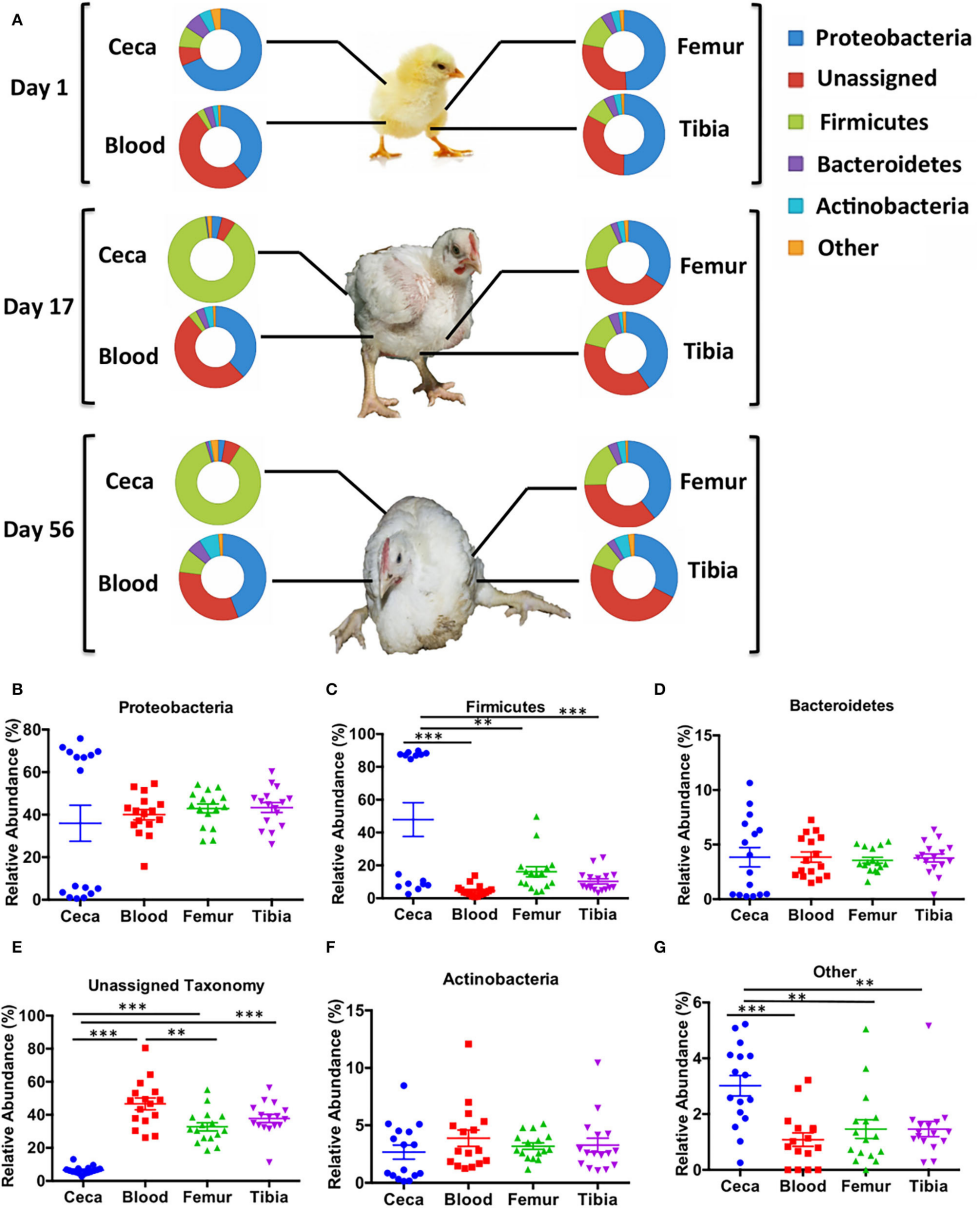
Taxonomic assignment of bacterial phyla in different sites of chicken. An operational taxonomic unit (OTU) table was normalized with the cumulative sum scaling (CSS) algorithm. **(A)** The relative abundance of bacterial phyla across ages (days 1, 17, and 56) and body sites (ceca, blood, femur, and tibia). **(B–G)** The overall relative abundance of bacterial phyla in different sites (ceca, blood, femur, and tibia) of chickens with combined ages. Data are mean ± standard error (S.E.). Samples were analyzed by ANOVA and Tukey's multiple-comparison test. **p < 0.01, ***p < 0.001.

Major bacterial phyla abundant in chicken ceca and extraintestinal sites (blood, femur, and tibia) were *Proteobacteria, Firmicutes, Bacteroidetes*, and *Actinobacteria* (Figure 1A). The relative abundance of bacterial phyla was similar among the extraintestinal sites at days 1, 17, and 56. However, the bacterial community at the phylum level of ceca at day 1 changed noticeably on days 17 and 56 (Figure 1A).

### Relative Abundance of Unassigned Reads Is Significantly Higher in Extraintestinal Sites Than Ceca Across All Ages in Chickens

The relative abundance of bacterial phyla was combined across all ages to their respective sites (Figures 1B–G). There was no difference in relative abundance of *Proteobacteria, Bacteroidetes*, and *Actinobacteria* among ceca, blood, femur, and tibia across all ages (Figures 1B–F). On the contrary, *Firmicutes* was significantly higher in the ceca than the extraintestinal sites (Blood, Femur, and Tibia) (*p* < 0.01, Tukey's multiple-comparison test) as shown in Figure 1C. The bimodal distribution of *Proteobacteria* and *Firmicutes* was seen in ceca due to the development of a reciprocal relation between these two phyla as the age of chickens increased (Figures 1A–C). Most intriguingly, the relative abundance of unassigned OTUs was significantly higher in blood, femur, and tibia than ceca (*p* < 0.001, Tukey's multiple-comparison test) (Figure 1E). Blood also had significantly higher unassigned OTUs as compared to the femur (*p* < 0.01) but not to tibia (*p* > 0.05, Tukey's multiple-comparison test). Other remaining phyla were significantly higher in ceca as compared to blood, femur, and tibia (*p* < 0.01, Tukey's multiple-comparison test) indicating the overall higher diversity in ceca at phylum level (Figure 1G).

### Bacterial Diversity Is Comparatively Stable in the Extraintestinal Sites of Chickens Across the Ages

The relative abundances of bacteria at the family and genus levels were similar among extraintestinal sites (blood, femur, and tibia) while distinct from ceca, at all ages (days 1, 17, and 56) of chickens as shown in Supplementary Figures 1, 2, respectively. The composition of ceca at day 1 looked similar to all extraintestinal sites at the family level but changed substantially at days 17 and 56 (Supplementary Figure 1). Additionally, the relative distribution of the top 50 most abundant OTUs at different sites at day 1 (Supplementary Figure 3) was similar among all sites, while day 17 (Supplementary Figure 4) and day 56 (Supplementary Figure 5) showed more similar abundant OTUs were shared among extraintestinal sites (blood, femur, and tibia) than with ceca. A significant number of OTUs (*n* = 59) were shared among ceca, blood, femur, and tibia on day 1 among the top 200 OTUs from each group. In contrast, fewer OTUs were shared between tissues on day 17 (*n* =1) and day 56 (*n* =4) as shown in Supplementary Figures 6A–C. Only 1 OTU was shared between ceca samples at three different ages of chickens (Supplementary Figure 6D). However, a higher number of OTUs were shared among the different extraintestinal sites across all ages of chickens. Blood shared 68 OTUs (Supplementary Figure 6E) and femur and tibia shared 73 and 55 OTUs, respectively, across three different ages (Supplementary Figures 6F,G). A similar bacterial composition at phylum, family, and genus levels and highly shared numbers of OTUs among the extraintestinal sites at all ages denote a relatively stable bacterial community of extraintestinal sites as opposed to the dynamic changes in the bacterial community in ceca during the transition from days 1 to 17.

**Figure 2:**
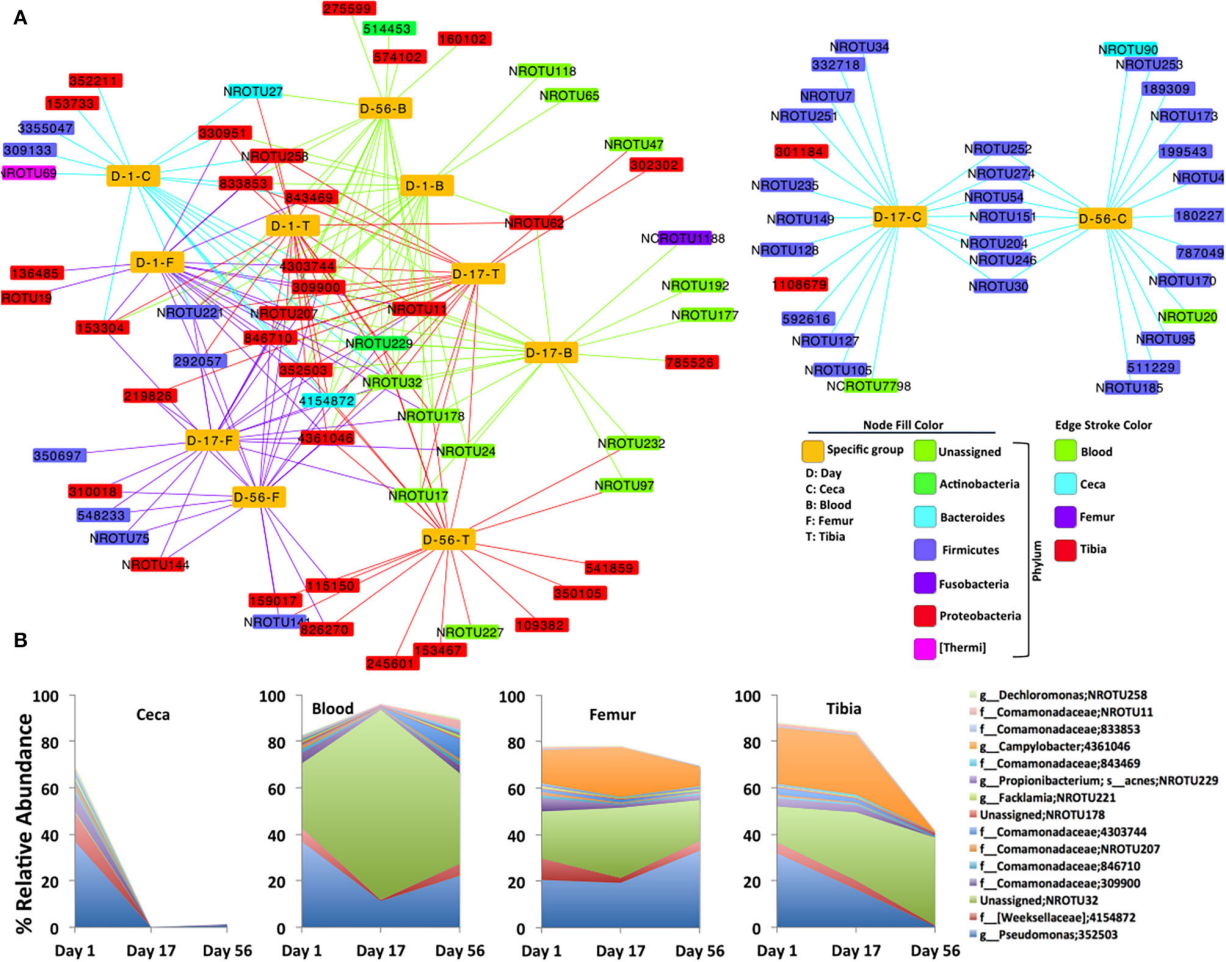
Bacterial OTUs interaction network. **(A)** Interaction of the top 20 abundant OTUs (small rectangular node) and each specific group of samples (yellow large rectangular node). Bacterial OTUs frequently shared by the specific group of chickens are at the core of the network, while uniquely present in any specific group of chickens, are toward the periphery of the network. **(B)** Area plot indicates the relative abundance of 15 most frequent OTUs that are at least shared by six specific groups of chickens (yellow node) in the bacterial network. Highest taxonomic rank is followed by OTU. NR: new reference, NCR: new cleanup reference, s, species; g, genus; f, family.

Furthermore, a bacterial OTU interaction network with the top 20 abundant OTUs of each site at each day point (4 sites X 3 days = 12 sample types) showed highly shared OTUs among all sites (ceca, blood, femur, and tibia) at day 1 and extraintestinal sites (blood, femur, and tibia) at day 17 and day 56 as shown in Figure 2A. The majority of shared OTUs belonged to either *Proteobacteria* or unassigned taxonomy. Intriguingly, none of the samples at day 1 and extraintestinal sites at days 17 and 56 shared the top 20 OTUs of ceca at days 17 and 56, most of which belonged to *Firmicutes* as shown by two distinct clusters of the network (Figure 2A). Notably, the interaction network of 15 most frequent OTUs that are shared among the 12 sample types showed a sharp decline in abundance in ceca at days 17 and 56 compared to day 1 but even abundance across all ages in blood, femur, and tibia (Figure 2B; see Supplementary File for representative DNA sequence of these most abundant OTUs). This network analysis additionally supports the quite stable bacterial community composition in the extraintestinal sites of chickens across different ages.

### Analyses of Alpha and Beta Diversity

**Figure 3:**
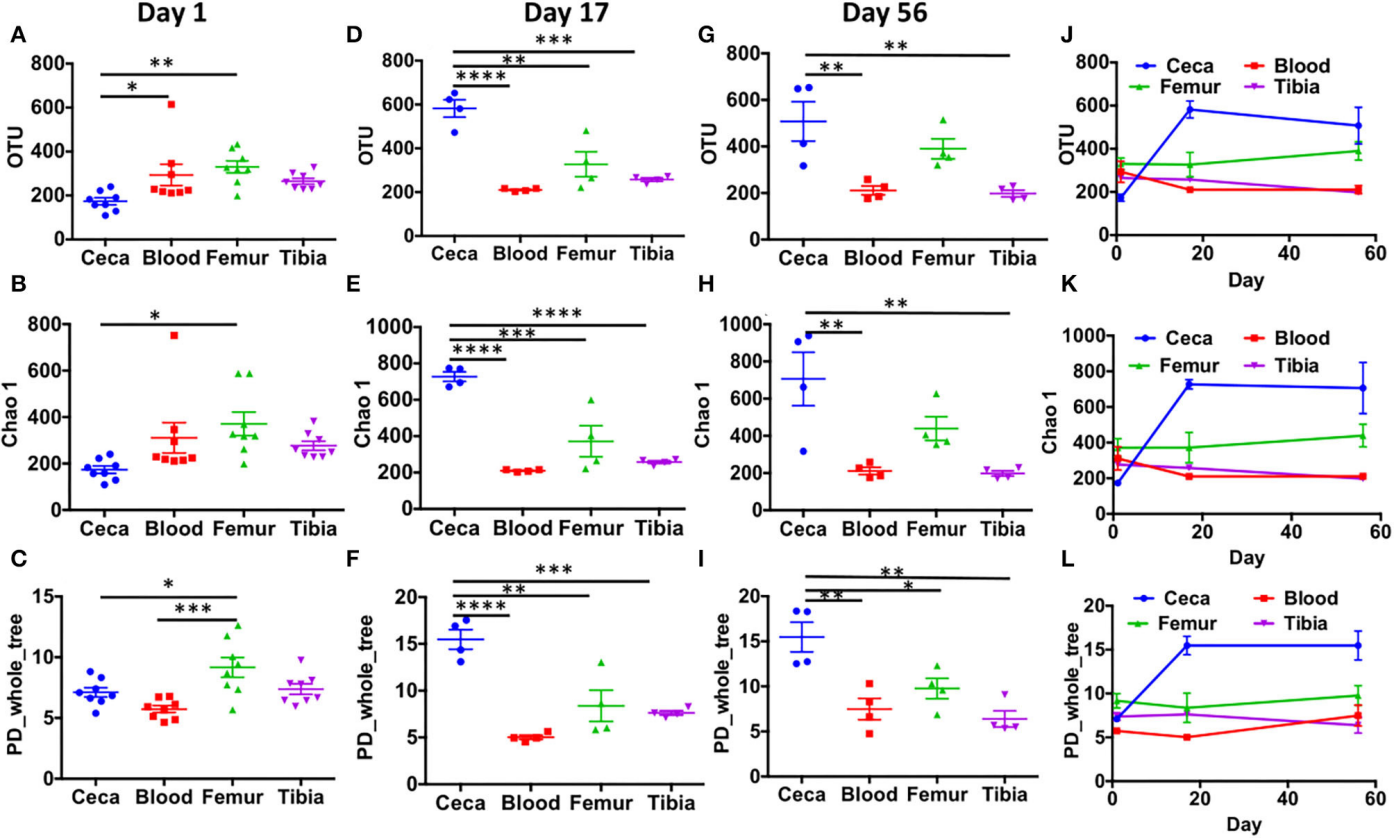
Alpha diversity in ceca, blood, femur, and tibia of chickens. CSS normalized OTU table was used to calculate alpha diversity indices like number of observed OTUs (OTU), chao1, and phylogenetic distance whole tree (PD_whole_tree). Bacterial diversity in day 1 **(A–C)**, day 17 **(D–F)**, and day 56 **(G–I)** across the different sites (ceca, blood, femur, and tibia). **(J–L)** The trend in bacterial diversity within different sites across the ages (days 1, 17, and 56) of chickens. Data are mean ± standard error (S.E.). Samples were analyzed by ANOVA and Tukey's multiple-comparison test. *p < 0.05, **p < 0.01, ***p < 0.001, ****p < 0.0001.

Alpha diversity analysis at the OTU level showed that extraintestinal sites (blood, femur, and tibia) had a similar number of observed OTUs, Chao1 values, and PD_whole_tree values at day 1 (Figures 3A–C), day 17 (Figures 3D–F), and day 56 (Figures 3G–I) (*p* > 0.05) with one exception that at day 1, PD_whole_tree of the femur was significantly higher than blood (Figure 3C; *p* < 0.001). Surprisingly, cecal samples had significantly lower alpha diversity compared to the femur at day 1 (*p* < 0.05; Figures 3A–C). The number of OTUs was also significantly higher in blood than ceca at day 1 (Figure 3A; *p* < 0.05). With the increase in age (days 17 and 56), cecal samples had significantly higher alpha diversity than extraintestinal sites (Figures 3D–I). There was a sharp increase in the alpha diversity indices of ceca from days 1 to 17 that remained flat through day 56 (Figures 3J–L). Intriguingly in our wire floor stress model, extraintestinal sites (blood, femur, and tibia) had stable alpha diversity with slight change over aging (Figures 3J–L).

**Figure 4:**
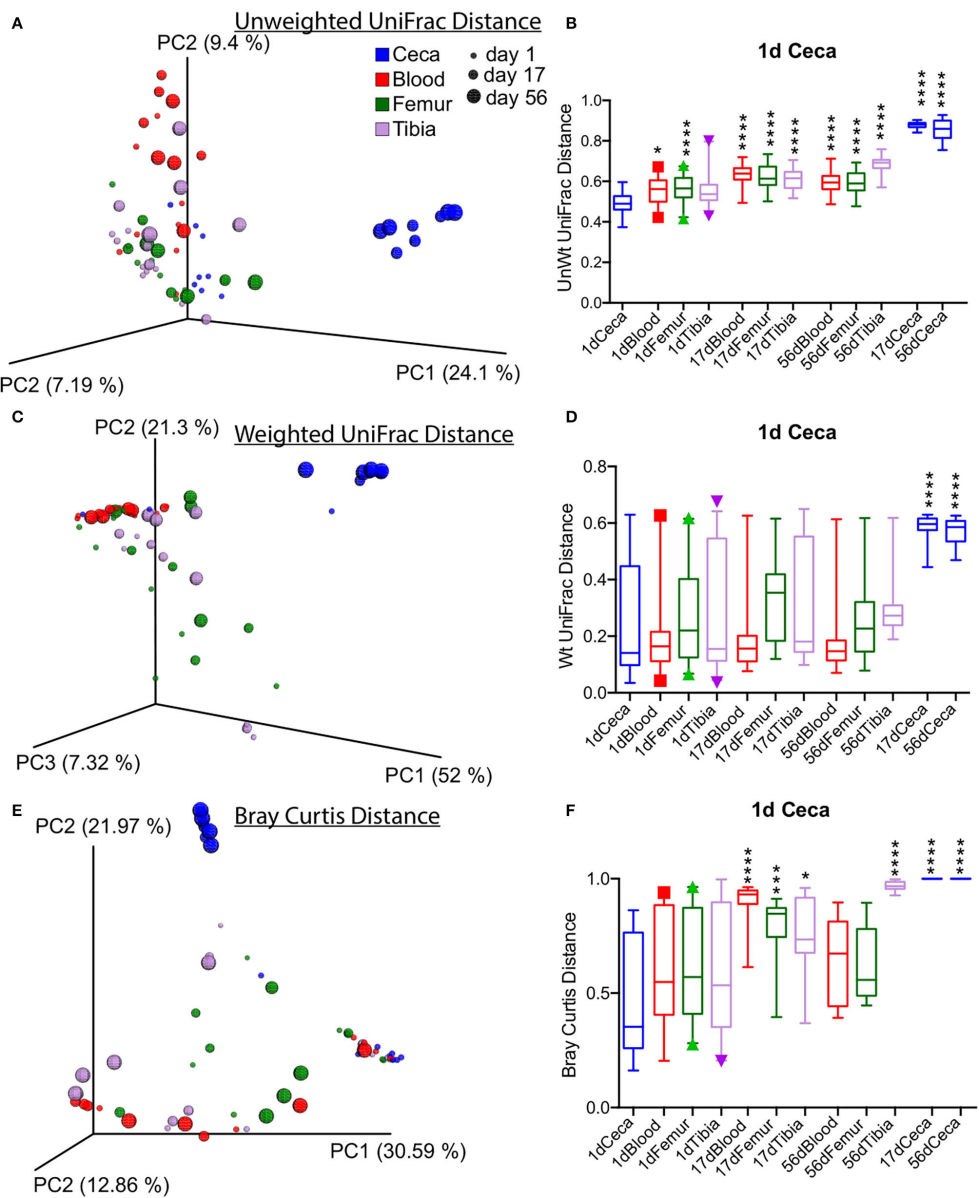
Overall beta diversity across sites and ages of chickens. **(A,C,E)** Principal coordinate analysis (PCoA) plots of different sites of chickens measured by unweighted, weighted UniFrac, and Bray Curtis dissimilarity distance at an even subsampling depth of 600 sequences/sample. **(B,D,F)** Distance of ceca at day 1 to respective sites. Boxplot displays median with whisker representing 2.5–97.5 percentile. Samples were analyzed by *t*-test with Bonferroni correction. *p < 0.05, ***p < 0.001, ****p < 0.0001.

Beta diversity analysis performed with unweighted and weighted UniFrac and Bray Curtis dissimilarity distance showed the distinct clustering of cecal samples at days 17 and 56 separately from the rest of the samples as shown by the PCoA plot (Figures 4A–F). The unweighted UniFrac distance of Ceca at day 1 was significantly different from the rest of extraintestinal sites and ceca at days 17 and 56 (Figure 4B). However, the weighted UniFrac distance of ceca at day 1 was only significantly different from ceca at days 17 and 56 (*p* < 0.0001, *t*-test; Figure 4D). Bray Curtis dissimilarity distance of ceca at day 1 was significantly different from blood and bone tissues at day 17, tibia at day 56, and ceca at day 17 and day 56 (*p* < 0.05, *T*-Test; Figure 4C). Largely, these data suggest the sharp divergence of cecal bacterial communities from the extraintestinal sites and day 1 ceca as the age increases (Figure 4).

Furthermore, an analysis of similarities (ANOSIM) indicated that age (*r* = 0.359 and *p* = 0.001) is as a stronger contributor of bacterial diversity than body sites (*r* = 0.272 and *p* = 0.001). Additional beta diversity analysis was performed on stratified data based on sites (ceca, blood, tibia, and femur). The PCoA plot revealed a distinct tight clustering of ceca according to different ages, and day 1 ceca was significantly different from days 17 and 56 ceca (*p* < 0.0001) (Supplementary Figures 7A–C). On the contrary, only a loose clustering of samples was seen in femur and tibia microbiota across different ages, but not in blood microbiota, as measured by unweighted UniFrac distances (Supplementary Figures 7F–L). UniFrac distance analysis showed that tibia microbiota at day 56 were different from day 1 (*p* < 0.001) and day 17 (*p* < 0.01), which might be due to development of BCO at day 56 (Supplementary Figures 7J–L). Also, the bacterial composition of femur at day 1 was different from day 17 (*p* < 0.05) with unweighted UniFrac distance (Supplementary Figures 7G–I). These analyses demonstrate that extraintestinal sites of chickens have similar bacterial community composition regardless of age and sites with a few exceptions.

### Gut May Be a Potential Source of Microbiota in Extraintestinal Sites of Chickens

**Figure 5:**
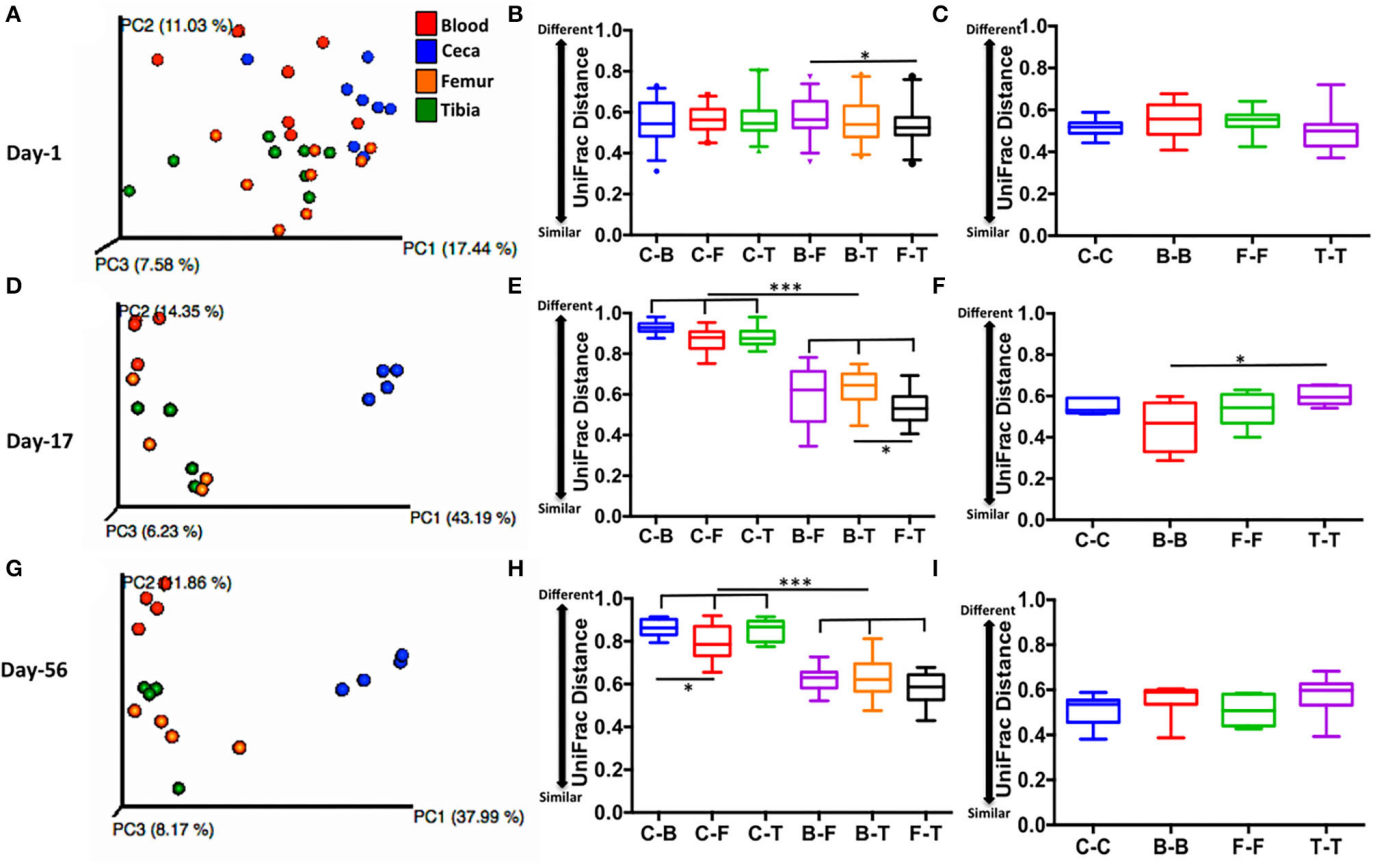
Level of similarity between sites measured by unweighted UniFrac distance. **(A,D,G)** PCoA plots of sites at days 1, 17, and 56 respectively. **(B,E,H)** Inter-site unweighted UniFrac distance at days 1,17, and 56, respectively. **(C,F,I)** Intra-site unweighted UniFrac distance at days 1, 17, and 56, respectively. UniFrac distance is based on subsampling depth of 600 sequences/sample. Unweighted UniFrac constituted both intra- and inter-subject distance. Boxplot displays median with whisker representing 2.5–97.5 percentile. Data were analyzed by ANOVA and Tukey's multiple-comparison test. Sites: C, ceca; B, blood; F, femur; and T, Tibia. *p < 0.05, ***p < 0.001.

Lower UniFrac distance indicates more similar bacterial composition between the samples. For example, 0 means 100% identical and 1 means 0% identical bacterial composition between the samples. PCoA plot displays scattered ceca, blood, femur, and tibia samples at day 1 (Figure 5A), while clustered ceca samples, at day 17 (Figure 5D) and day 56 (Figure 5G) distinctively from extraintestinal sites (blood, femur, and tibia). Interestingly, a lower inter-site UniFrac distance of ceca to blood (C-B), femur (C-F), and tibia (C-T) were observed on day 1. Inter-site UniFrac distances at day 1 (C-B, C-F, and C-T; Figure 5B) were comparable to the intra-site distance of ceca, blood, femur, and tibia (C-C, B-B, F-F, and T-T; Figure 5C) but not at day 17 (Figures 5E,F), and day 56 (Figures 5H,I). This indicates more similar bacterial composition at day 1 between ceca and extra-intestinal sites. However, at days 17 and 56, the inter-site UniFrac distance of ceca to blood (C-B), femur (C-F), and tibia (C-T) was >0.8 (indicating 80% dissimilar) and was significantly different from the B-F, B-T, and F-T at day 17 (Figure 5E) and day 56 (Figure 5H) (*p* < 0.001). This indicates more dissimilar bacterial composition between ceca and extraintestinal sites at day 17 and day 56. Taken together, these data suggest that ceca (gut) at day 1 may be the potential source of microbiota in extraintestinal sites.

**Figure 6:**
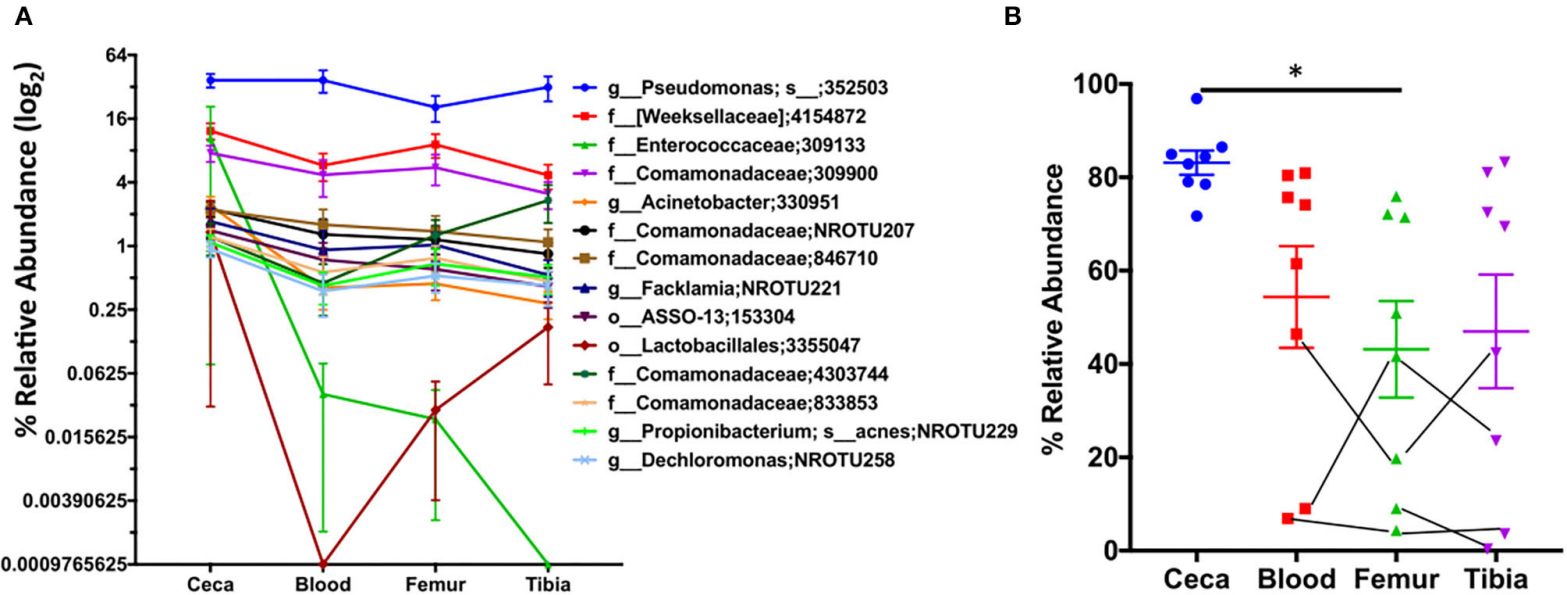
Spatiotemporal distribution of top 15 OTUs of ceca. The relative abundance of the top 15 OTUs of ceca at day 1 and their relative abundance in blood, femur, and tibia at day 1. **(A)** The relative abundance of top 15 OTUs of ceca (source) into the extraintestinal site (sink: blood, femur, and tibia) of chickens. Each point is the mean relative abundance (±S.E.) of eight chickens. **(B)** The cumulative abundance of the aforementioned 15 OTUs. Lines connecting the dots are the same chicken. Data are mean ± S.E. Samples were analyzed by ANOVA and Tukey's multiple-comparison test. *p < 0.05.

Next, we examined the relative abundance of top 15 OTUs in ceca at day 1 and their abundance in extraintestinal sites of chickens (Figure 6). We reason that spatial distribution of these abundant OTUs at day 1 should show higher abundance in ceca (source) as compared to extraintestinal sites (sink) in the absence of a long temporal gap (age of chickens). Interestingly, a decreasing trend in the relative abundance of majority of the top 15 OTUs was observed from ceca to blood to bones (Figure 6A). Additionally, the relative abundance of these top 15 OTUs in the ceca at day 1 was significantly lower in femur than in ceca (Figure 6B), nonetheless still preserving the decreasing trend of bacterial abundance from source (ceca) to sink (extraintestinal sites). Thus, these data may suggest the gut as a potential source of microbiota in extraintestinal sites and probability of unidirectional translocation of microbiota from gut to the extraintestinal site.

### Probable Bacteria Able to Translocate Across the Impaired Gut Barrier Due to Stress

**Figure 7:**
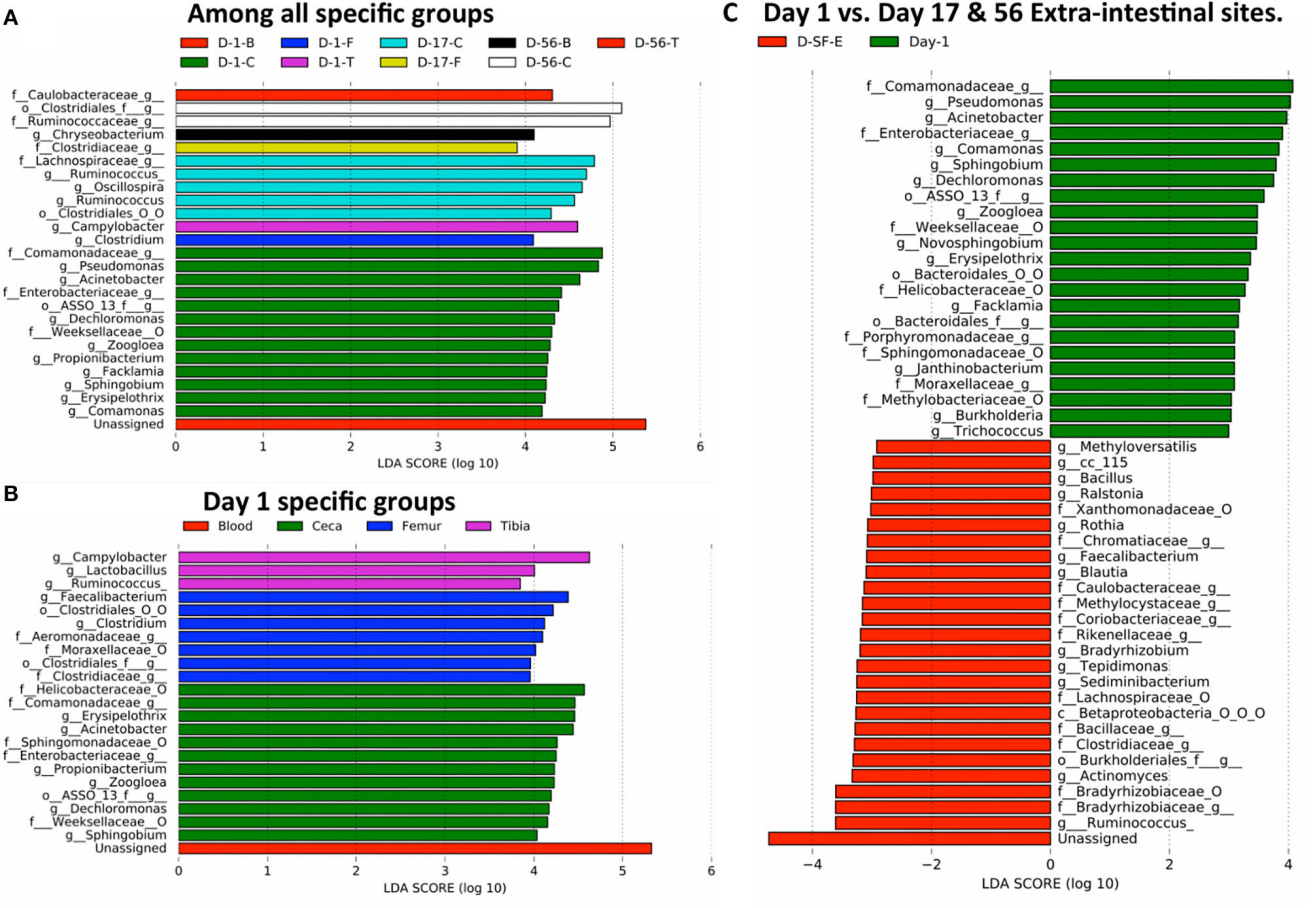
Translocation of potential bacteria from gut to extra-intestinal sites of chickens. The taxonomic abundance table at the genus level was used for the LEfSe analysis. Differentially abundant taxonomic features among: **(A)** specific groups (4 sites × 3 days = 12 specific groups/sample types) of entire study, **(B)** day 1 specific groups (blood, ceca, femur, and tibia), and **(C)** day 1 (all 4 sites) vs. days 17 and 56 extraintestinal sites (D-SF-E). f__, family; g__, genus; o__, order; O, other, and the taxonomic level is connected by an underscore (_).

Finally, we investigated probable bacteria able to translocate from the ceca to extraintestinal sites due to stress caused by wire flooring. Linear discriminant analysis effect size (LEfSe) was performed to identify the differentially abundant bacteria among different sample types at their lowest taxonomic assignment with 97% sequence similarity. Firstly, bacteria that are enriched in each of 12 specific groups (3 days × 4 sites = 12 groups) were examined (Figure 7A and Supplementary Figure 8). None of the bacteria was differentially abundant in blood and tibia at day 17 and femur at day 56. The majority of bacteria differentially abundant were present in the ceca at different ages of chickens, accounting for 20 out of 26 features. *Clostridium* and *Campylobacter* were differentially abundant in the femur and tibia on day 1, respectively (Figure 7A and Supplementary Figure 8A). *Clostridiaceae, Chryseobacterium* (Supplementary Figure 8B), and *Caulobacteriaceae* were significantly enriched in the femur (day 17), blood (day 56), and tibia (day 56), respectively (Figure 7A).

Moreover, differentially abundant bacteria were analyzed among the four different sites on day 1. Unassigned taxonomy was differentially abundant in blood. The cecum was enriched with six genera (*Sphingobium, Dechloromonas, Zoogloea, Propionibacterium, Acinetobacter*, and *Erysipelothrix*), femur with two genera (*Clostridium* and *Faecalibacterium*), and tibia with three genera (*Ruminococcus, Lactobacillus*, and *Campylobacter*) (Figure 7B and Supplementary Figure 9). Thus, the differential abundance of distinct bacteria among four sites on day 1 may suggest the existence of site-specific intrinsic selective pressures even in 1 day-old chickens.

Earlier, we showed that the extraintestinal sites of chickens have a relatively unchanged bacterial composition across the ages, and all the sites on day 1 have a mostly similar bacterial composition. Therefore, we reason that any changes in microbial community composition in extraintestinal sites on days 17 and 56 as compared to all sites on day 1 would be considered due to additional translocation from the gut through epithelial barrier after day 1 and/or differential multiplication of bacteria translocated on day 1 according to the distinct selective pressures in each extraintestinal site over the following days (Figure 7C). Bacterial genera that might have crossed the gut barrier due to stress were *Methyloversaltilis, Bacillus, Ralstonia, Rothia, Faecalibacterium, Blautia, Bradyrhizobium, Tepidimonas, Sediminibacterium, Actinomyces*, and *Ruminococcus* (Figure 7C and Supplementary Figure 10). Interestingly, *Ruminococcus* was enriched in ceca at day 17 (Figure 7A) and tibia at day 1 (Figure 7B). Overabundance of *Ruminococcus* in days 17 and 56 extraintestinal sites as compared to day 1 sites might be due to the growth of *Ruminococcus* already present in the tibia or leakage of the bacterium from ceca at day 17 (Figure 7C). Hence, differentially abundant bacteria in extraintestinal sites at days 17 and 56 could be either due to translocation from the gut or multiplication of bacteria that were already present in extraintestinal sites on day 1. On the other hand, *Pseudomonas, Acinetobacter, Comamonas, Sphingobium*, and *Dechloromonas* were the most abundant genera at day 1 including others as compared to extraintestinal sites at days 17 and 56 (Figure 7C).

## Discussion

In this study, gut (ceca) and extraintestinal site (blood, femur, and tibia head) microbiota of chickens raised on the wire floor were explored to reveal the composition of microbiota and source of microbiota in extraintestinal sites using 16S rRNA gene sequencing. To note, all of the chickens sampled at day 56 had BCO, while the other time points were healthy.

Surprisingly, extraintestinal sites had a significantly higher relative abundance of unassigned reads than ceca regardless of age and stress (1). These reads might indicate novel taxa (OTUs) that still need to be characterized. Very few unassigned reads in ceca negate the possibility of chimera formation and error in the bioinformatics pipeline because of highly characterized gut microbiota. The result is consistent with the previous study where we had found >70% of unassigned reads in the chicken blood (16). In the same line, Kowarsky et al. (25) used the shotgun metagenome sequence to characterize the blood microbiota for human patients and identified >50% of novel contigs having coding sequence with little to no sequence homology in the existing database. Additionally, only 285 (14.2%) of 2006 unassigned OTUs significantly matched to the chicken reference genome. Minimum, median, and maximum relative abundance of OTUs in extra-intestinal sites that matched significantly to the chicken genome were 0.08, 21.65, and 93.3%, respectively (Supplementary Figure 11). Filtering out these significantly matched OTUs to host genome had very minimal impact on alpha and beta diversity indices as well as on relative abundance of unassigned reads (Supplementary Figure 12).

Blood circulates throughout the body of animals; thus, it is expected that microbes in the blood can reach all tissues and organs in the body, though they might be subsequently subjected to the selective pressure according to the unique microenvironments in the particular body sites. These unique microenvironments between mature ceca (on days 19 and 56) and extraintestinal sites as well as undeveloped ceca (day 1) might contribute to the bimodal distribution of *Firmicutes* and *Proteobacteria*. Interestingly, however, we observed similar bacterial composition among all sites on day 1 and extraintestinal sites on days 17 and 56 compared to ceca (days 17 and 56) as indicated by taxonomic assignment, OTU interaction network, and alpha and beta diversity analysis. Lower UniFrac distance from ceca to extraintestinal sites (blood, femur and tibia) and spatiotemporal distribution of microbiota at day 1 may suggest the gut as the source of extraintestinal microbiota (Figures 5, 6). Ilina et al. (26) discovered 38 phylotypes of microbiota were able to colonize the gastrointestinal tract of the chicken embryo using terminal restriction fragment length polymorphism (T-RFLP). However, higher alpha diversity index (OTUs, Chao 1 and PD_whole_tree) in the extraintestinal site of chicks than ceca at day 1 portrays a different story. There is the possibility that *in ovo* microbiota (non-sterile womb) colonization of ovarian follicles and/or that bacterial translocation from maternal circulation might be the source of intestinal and extraintestinal microbiota in 1 day-old chickens (27). Recent studies have identified bacteria in the endometrium, placenta, amniotic fluid, and meconium from healthy pregnancies in humans, which suggests a non-sterile womb (28–31). The non-sterile uterine microbiota may diffuse and/or translocate from maternal circulation to ovarian follicles. Later during embryogenesis and growth, microbiota are selected by tissue- and organ-specific niches to become the resident microbiota. In a recent study, Lee et al. (32) detected 21 core genera of bacteria in chicken magnum, cloaca, descendent egg shell, egg white, and embryo gut. The authors also reported that maternal oviduct microbiota was the source of embryo gut microbiota.

When the chickens were raised on the wire floor, a significantly higher number of birds develop lameness due to BCO (18). All the birds sampled at day 56 in this study were lame. LEfSe analysis revealed differentially abundant bacteria in extraintestinal sites on day 17 and 56 as compared to day 1 microbiota (ceca and extraintestinal), which may indicate differential selective pressures imposed on bacteria in extraintestinal sites as the age of chickens increases or translocation from the gut. Both pathogenic and commensal bacteria were able to cross the impaired gut epithelial barrier, which may have caused by stress (Figure 7). *Actinomyces* spp. normally colonizes the human mouth, digestive, and genital tracts and is an infrequent cause of invasive bacterial disease of bone and joints including others (33). *Sediminibacterium* has been isolated from liver abscess (34). Similarly, *Tepidomonas* spp. has been isolated from the bone marrow of a patient with leukemia in Korea (35). *Blautia* spp. is one of the abundant groups of the human gastrointestinal tract and provides energy to their host by metabolizing polysaccharide that other gut bacteria cannot degrade (36). *Blautia* spp. is positively correlated with intestinal permeability in alcoholic liver disease (37). *Rothia* has been isolated from osteomyelitis and joint lesions in turkeys. However, *Bacillus* and *Faecalibacterium* spp. have anti-inflammatory activity and seem to antagonize the other pathogenic bacteria in the bone and femur of BCO chickens (38, 39).

The study had some limitations in that the study lacked appropriate control i.e., chickens raised in non-stressed conditions. Due to the low biomass of microbiota in extraintestinal sites, the possibility of contamination from kits were not ruled out. Nonetheless, no visible PCR amplification band was seen in PCR water negative control samples. In addition, our previous study analyzing microbiota showed exclusively tight clustering between the right and left femur or tibia bones from the same individual chickens (17). The result strongly suggests that the microbiota profiles in the current study in which the same sample processing and DNA extraction methods were used reflect the microbiota in the collected samples, rather than contaminating bacteria or DNA.

In summary, this is the first comprehensive study in any vertebrate animals to our knowledge that looked into the relationship among microbiota in the gut and three extraintestinal organs at three different life stages.

## Conclusion

In conclusion, we comprehensively characterized microbiota of chickens raised under stressed conditions. The abundance of novel taxa in the extraintestinal sites needs to be further explored for their role in the health and disease of chickens and other animals in large and high-powered studies. Ultimately, our findings will help in the efficient management of chicken production, health, and welfare issues.

## Data Availability Statement

The assembled paired-end chimera-removed reads have been deposited at DDBJ/ENA/GenBank under the accession KCET000000000 as Targeted Locus Study project. The version described in this paper is the first version, KCET010000000 and is available under this link https://www.ncbi.nlm.nih.gov/nuccore/KCET000000000.1.

## Ethics Statement

The animal study was reviewed and approved by University of Arkansas Institutional Animal Care and Use Committee.

## Author Contributions

RW, TL, and YK designed the overall study. RM and TJ performed experiments. RM, TJ, and YK analyzed microbiota data. RM and YK wrote the manuscript. All authors contributed to the article and approved the submitted version.

## Conflict of Interest

TL was employed by Quality Technology International (QTI), Inc. The remaining authors declare that the research was conducted in the absence of any commercial or financial relationships that could be construed as a potential conflict of interest. The authors also declare that this study received funding from QTI. The funder was not involved in the study design, collection, analysis, interpretation of data, the writing of this article or the decision to submit it for publication.

## References

[1] Schneiderman N Ironson G Siegel SD. Stress and health: psychological, behavioral, and biological determinants. Annu Rev Clin Psychol. (2005) 1:607–28. 10.1146/annurev.clinpsy.1.102803.144141 17716101 PMC2568977

[2] de Punder K Pruimboom L. Stress induces endotoxemia and low-grade inflammation by increasing barrier permeability. Front Immunol. (2015) 6:223. 10.3389/fimmu.2015.00223 26029209 PMC4432792

[3] Vanuytsel T van Wanrooy S Vanheel H Vanormelingen C Verschueren S Houben E. Psychological stress and corticotropin-releasing hormone increase intestinal permeability in humans by a mast cell-dependent mechanism. Gut. (2014) 63:1293–9. 10.1136/gutjnl-2013-305690 24153250

[4] Costa FR Francozo MC de Oliveira GG Ignacio A Castoldi A Zamboni DS. Gut microbiota translocation to the pancreatic lymph nodes triggers NOD2 activation and contributes to T1D onset. J Exp Med. (2016) 213:1223–39. 10.1084/jem.20150744 27325889 PMC4925011

[5] Giannelli V Di Gregorio V Iebba V Giusto M Schippa S Merli M. Microbiota and the gut-liver axis: bacterial translocation, inflammation and infection in cirrhosis. World J Gastroenterol. (2014) 20:16795–810. 10.3748/wjg.v20.i45.16795 25492994 PMC4258550

[6] Lelouvier B Servant F Païssé S Brunet A Benyahya S Serino M. Changes in blood microbiota profiles associated with liver fibrosis in obese patients: a pilot analysis. Hepatology. (2016) 64:2015–27. 10.1002/hep.28829 27639192

[7] von Klitzing E Ekmekciu I Kühl AA Bereswill S Heimesaat MM. Intestinal, extra-intestinal and systemic sequelae of *Toxoplasma gondii* induced acute ileitis in mice harboring a human gut microbiota. PLoS ONE. (2017) 12:e0176144. 10.1371/journal.pone.0176144 28414794 PMC5393883

[8] Mirza A Mao-Draayer Y. The gut microbiome and microbial translocation in multiple sclerosis. Clin Immunol. (2017) 183:213–24. 10.1016/j.clim.2017.03.001 28286112

[9] Stanley D Mason LJ Mackin KE Srikhanta YN Lyras D Prakash MD. Translocation and dissemination of commensal bacteria in post-stroke infection. Nat Med. (2016) 22:1277–84. 10.1038/nm.4194 27694934

[10] Marchetti G Tincati C Silvestri G. Microbial translocation in the pathogenesis of HIV infection and AIDS. Clin Microbiol Rev. (2013) 26:2–18. 10.1128/CMR.00050-12 23297256 PMC3553668

[11] Vrakas S Mountzouris KC Michalopoulos G Karamanolis G Papatheodoridis G Tzathas C. Intestinal bacteria composition and translocation of bacteria in inflammatory bowel disease. PLoS ONE. (2017) 12:e0170034. 10.1371/journal.pone.0170034 28099495 PMC5242456

[12] Slyepchenko A Maes M Jacka FN Kohler CA Barichello T McIntyre RS. Gut microbiota, bacterial translocation, and interactions with diet: pathophysiological links between major depressive disorder and non-communicable medical comorbidities. Psychother Psychosom. (2017) 86:31–46. 10.1159/000448957 27884012

[13] Bangsgaard Bendtsen KM Krych L Sorensen DB Pang W Nielsen DS Josefsen K. Gut microbiota composition is correlated to grid floor induced stress and behavior in the BALB/c mouse. PLoS ONE. (2012) 7:e46231. 10.1371/journal.pone.0046231 23056268 PMC3462757

[14] Chen J Tellez G Richards JD Escobar J. Identification of potential biomarkers for gut barrier failure in broiler chickens. Front Vet Sci. (2015) 2:14. 10.3389/fvets.2015.00014 26664943 PMC4672187

[15] Kuttappan VA Vicuna EA Latorre JD Wolfenden AD Tellez GI Hargis BM. Evaluation of gastrointestinal leakage in multiple enteric inflammation models in chickens. Front Vet Sci. (2015) 2:66. 10.3389/fvets.2015.00066 26697435 PMC4677096

[16] Mandal RK Jiang T Al-Rubaye AA Rhoads DD Wideman RF Zhao J. An investigation into blood microbiota and its potential association with bacterial chondronecrosis with osteomyelitis (BCO) in Broilers. Sci Rep. (2016) 6:25882. 10.1038/srep25882 27174843 PMC4865835

[17] Jiang T Mandal RK Wideman RF Jr. Khatiwara A Pevzner I Kwon YM. Molecular survey of bacterial communities associated with bacterial chondronecrosis with osteomyelitis (BCO) in broilers. PLoS ONE. (2015) 10:e0124403. 10.1371/journal.pone.0124403 25881241 PMC4400152

[18] Wideman RF Jr Hamal KR Stark JM Blankenship J Lester H Mitchell KN. A wire-flooring model for inducing lameness in broilers: evaluation of probiotics as a prophylactic treatment. Poult Sci. (2012) 91:870–83. 10.3382/ps.2011-01907 22399726

[19] Gilley A Lester H Pevzner I Anthony N Wideman R Jr. Evaluating portable wire-flooring models for inducing bacterial chondronecrosis with osteomyelitis in broilers. Poult Sci. (2014) 93:1354–67. 10.3382/ps.2013-03781 24879685

[20] Caporaso JG Kuczynski J Stombaugh J Bittinger K Bushman FD Costello EK. QIIME allows analysis of high-throughput community sequencing data. Nat Methods. (2010) 7:335–6. 10.1038/nmeth.f.303 20383131 PMC3156573

[21] Edgar RC. Search and clustering orders of magnitude faster than BLAST. Bioinformatics. (2010) 26:2460–1. 10.1093/bioinformatics/btq461 20709691

[22] Segata N Izard J Waldron L Gevers D Miropolsky L Garrett WS. Metagenomic biomarker discovery and explanation. Genome Biol. (2011) 12:R60. 10.1186/gb-2011-12-6-r60 21702898 PMC3218848

[23] Li H Durbin R. Fast and accurate long-read alignment with Burrowsâ*e*“Wheeler transform. Bioinformatics. (2010) 26:589–95. 10.1093/bioinformatics/btp698 20080505 PMC2828108

[24] Shannon P Markiel A Ozier O Baliga NS Wang JT Ramage D. Cytoscape: a software environment for integrated models of biomolecular interaction networks. Genome Res. (2003) 13:2498–504. 10.1101/gr.1239303 14597658 PMC403769

[25] Kowarsky M Camunas-Soler J Kertesz M de Vlaminck I Koh W Pan W. Numerous uncharacterized and highly divergent microbes which colonize humans are revealed by circulating cell-free DNA. Proc Natl Acad Sci USA. (2017) 114:9623–8. 10.1073/pnas.1707009114 28830999 PMC5594678

[26] Ilina LA Yildirim EA Nikonov IN Filippova V Laptev G Novikova N et al. Metagenomic bacterial community profiles of chicken embryo gastrointestinal tract by using T-RFLP analysis. Dokl Biochem Biophys. (2016) 466:47–51. 10.1134/S1607672916010130 27025487

[27] Stinson LF Payne MS Keelan JA. Planting the seed: origins, composition, and postnatal health significance of the fetal gastrointestinal microbiota. Crit Rev Microbiol. (2017) 43:352–69. 10.1080/1040841X.2016.1211088 27931152

[28] Collado MC Rautava S Aakko J Isolauri E Salminen S. Human gut colonisation may be initiated *in utero* by distinct microbial communities in the placenta and amniotic fluid. Sci Rep. (2016) 6:23129. 10.1038/srep23129 27001291 PMC4802384

[29] Aagaard K Ma J Antony KM Ganu R Petrosino J Versalovic J The placenta harbors a unique microbiome. Sci Transl Med. (2014) 6:237ra65 10.1126/scitranslmed.3008599 PMC4929217 24848255

[30] Tao X Franasiak JM Zhan Y Scott RT Rajchel J Bedard J Characterizing the endometrial microbiome by analyzing the ultra-low bacteria from embryo transfer catheter tips in IVF cycles: next generation sequencing (NGS) analysis of the 16S ribosomal gene. Hum Microb J. (2017) 3:15–21. 10.1016/j.humic.2017.01.004

[31] Franasiak J Werner M Juneau C Tao X Landis J Zhan Y. Endometrial microbiome at the time of embryo transfer: next-generation sequencing of the 16S ribosomal subunit. J Assist Reprod Genet. (2016) 33:129–36. 10.1007/s10815-015-0614-z 26547201 PMC4717132

[32] Lee S La T Lee H Choi I Song C Park S. Characterization of microbial communities in the chicken oviduct and the origin of chicken embryo gut microbiota. Sci Rep. (2019) 9:6838. 10.1038/s41598-019-43280-w 31048728 PMC6497628

[33] Valour F Sénéchal A Dupieux C Karsenty J Lustig S Breton P. Actinomycosis: etiology, clinical features, diagnosis, treatment, and management. Infect Drug Resist. (2014) 7:183–97. 10.2147/IDR.S39601 25045274 PMC4094581

[34] Reyna-Fabian ME Zermeno V Ximenez C Flores J Romero MF Diaz D. Analysis of the Bacterial diversity in liver abscess: differences between pyogenic and amebic abscesses. Am J Trop Med Hyg. (2016) 94:147–55. 10.4269/ajtmh.15-0458 26572872 PMC4710420

[35] Ko KS Lee NY Oh WS Lee JH Ki HK Peck KR. *Tepidimonas arfidensis* sp. nov., a novel gram-negative and thermophilic bacterium isolated from the bone marrow of a patient with leukemia in Korea. Microbiol Immunol. (2005) 49:785–8. 10.1111/j.1348-0421.2005.tb03669.x 16113507

[36] Eren AM Sogin ML Morrison HG Vineis JH Fisher JC Newton RJ. A single genus in the gut microbiome reflects host preference and specificity. ISME J. (2015) 9:90–100. 10.1038/ismej.2014.97 24936765 PMC4274434

[37] Leclercq S Matamoros S Cani PD Neyrinck AM Jamar F Starkel P. Intestinal permeability, gut-bacterial dysbiosis, and behavioral markers of alcohol-dependence severity. Proc Natl Acad Sci USA. (2014) 111:E4485–93. 10.1073/pnas.1415174111 25288760 PMC4210345

[38] Sokol H Pigneur B Watterlot L Lakhdari O Bermudez-Humaran LG Gratadoux JJ. *Faecalibacterium prausnitzii* is an anti-inflammatory commensal bacterium identified by gut microbiota analysis of Crohn disease patients. Proc Natl Acad Sci USA. (2008) 105:16731–6. 10.1073/pnas.0804812105 18936492 PMC2575488

[39] Paynich ML Jones-Burrage SE Knight KL. Exopolysaccharide from *Bacillus subtilis* induces anti-inflammatory M2 macrophages that prevent T cell-mediated disease. J Immunol. (2017) 198:2689–98. 10.4049/jimmunol.1601641 28202619 PMC5360499

