# Supplementary material for: Microbiota Analysis of Chickens Raised Under Stressed Conditions
Source: Front Vet Sci. 2020 Oct 7;7:482637. doi: 10.3389/fvets.2020.482637 (PMC7575692; doi:10.3389/fvets.2020.482637)
Supplement: Supplementary file 1 [file Data_Sheet_1.PDF]

Supplementary File:

Microbiota analysis of chickens raised under stress condition

Rabindra K. Mandal[1,3*], Tieshan Jiang[1], Robert F. Wideman Jr.[1], Troy Lohrmann[4], and Young Min Kwon[1]

[1]Department of Poultry Science, University of Arkansas, Fayetteville, AR

[2]Cell and Molecular Biology Program, University of Arkansas, Fayetteville, AR

[3]Current Address: Department of Pediatrics, Indianapolis, IN

[4]Quality Technology International, Inc. Elgin, Illinois, USA

This file contains supplemental figures: 1-11, Supplemental Table 1 and 16S rRNA gene DNA sequences of top fifteen most frequent OTUs in this study from Fig 3B in FASTA format.

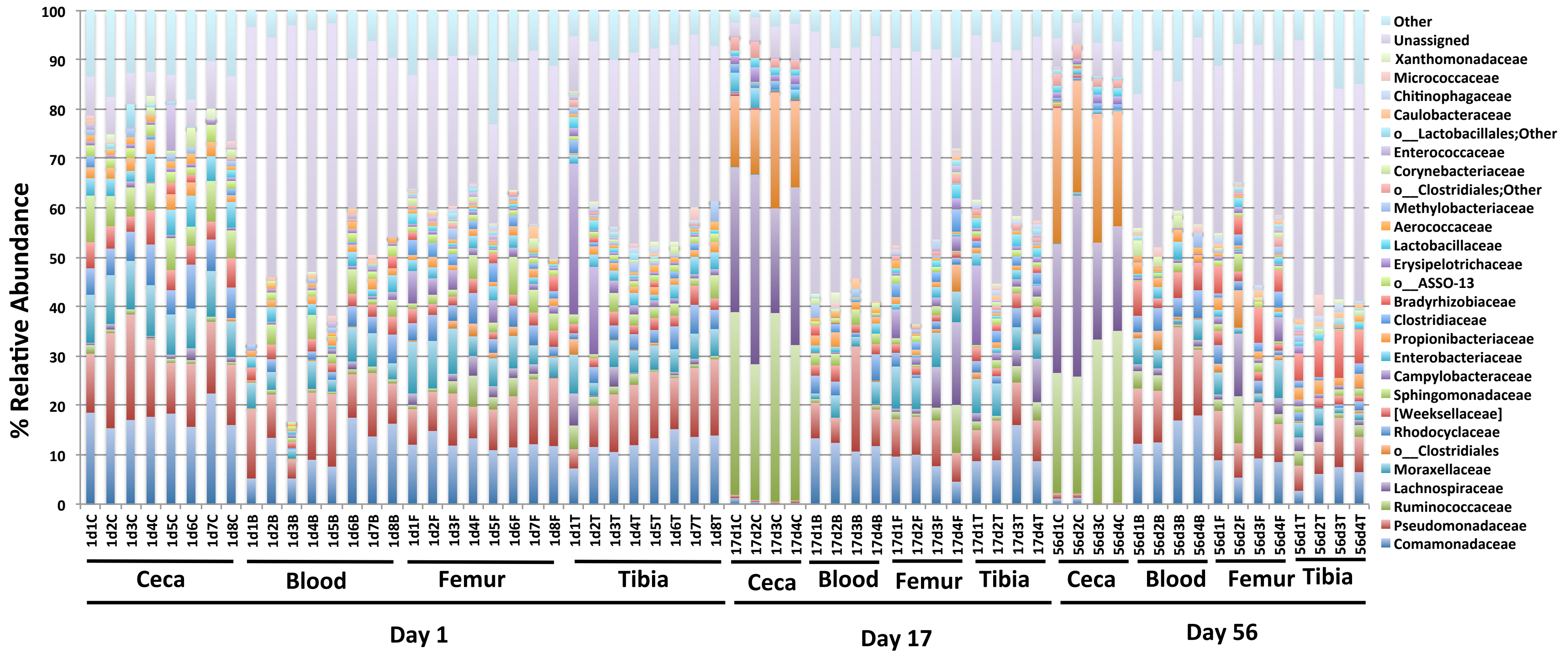

Supplemental Figure 1. Relative abundance of top 28 families. Other includes the sum of lower abundant families. Samples are named in the pattern as in 1d1C (which stands for 1d: day 1, followed by 1: chicken number 1, and C: ceca; and B: Blood, F: Femur, and T: Tibia).

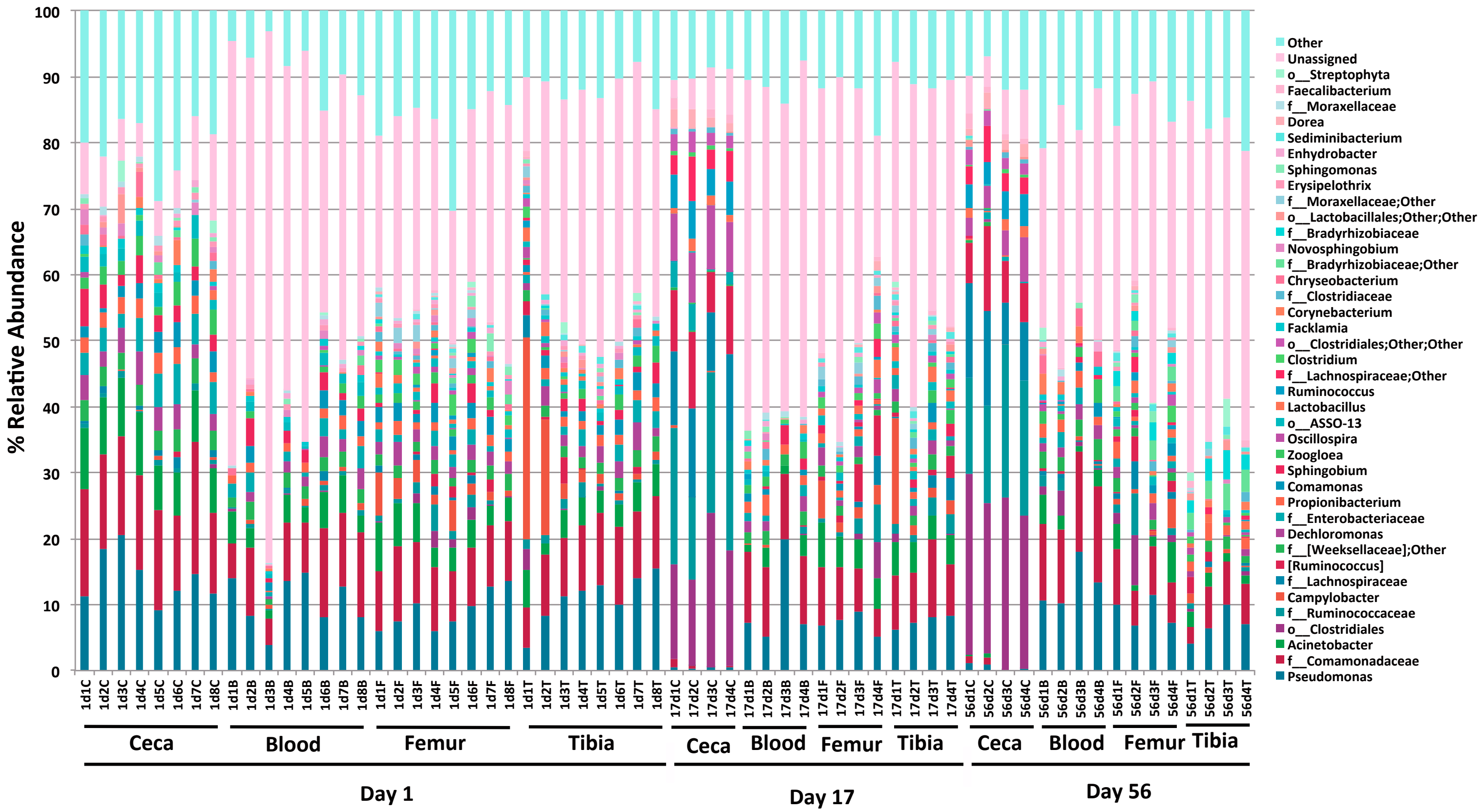

Supplemental Figure 2. Relative abundance of top 40 genera. Other includes the sum of lower abundant genera. Samples are named in the pattern as in 1d1C (1d: day 1, followed by 1: chicken number 1, and C: ceca, B: Blood, F: Femur, and T: Tibia).

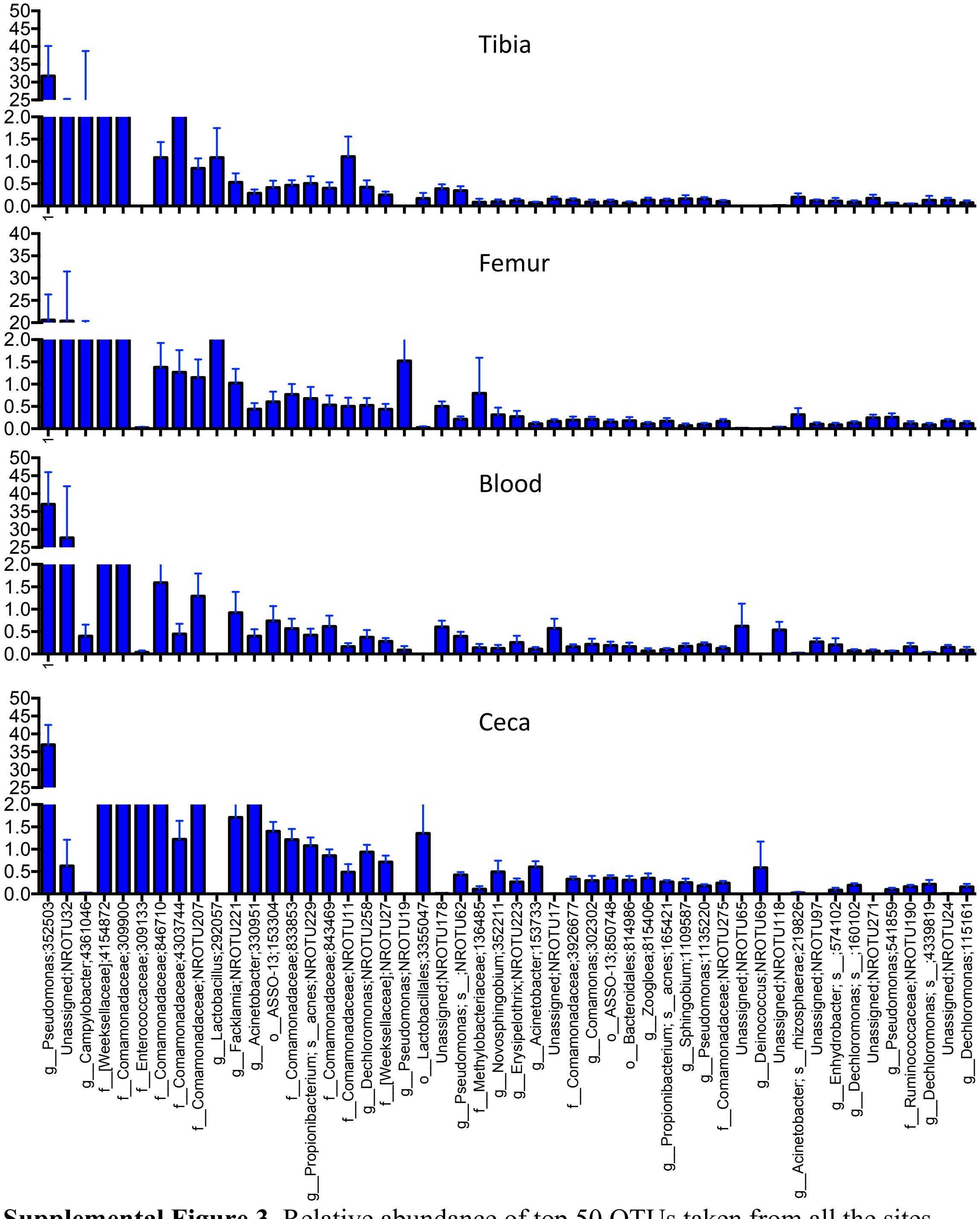

Supplemental Figure 3. Relative abundance of top 50 OTUs taken from all the sites combined on day 1. Bar depicts mean ± S.E. The highest taxonomic rank is shown followed by OTU number. O__: order, f__: family, g__: genus, s__: species, and NR-new reference.

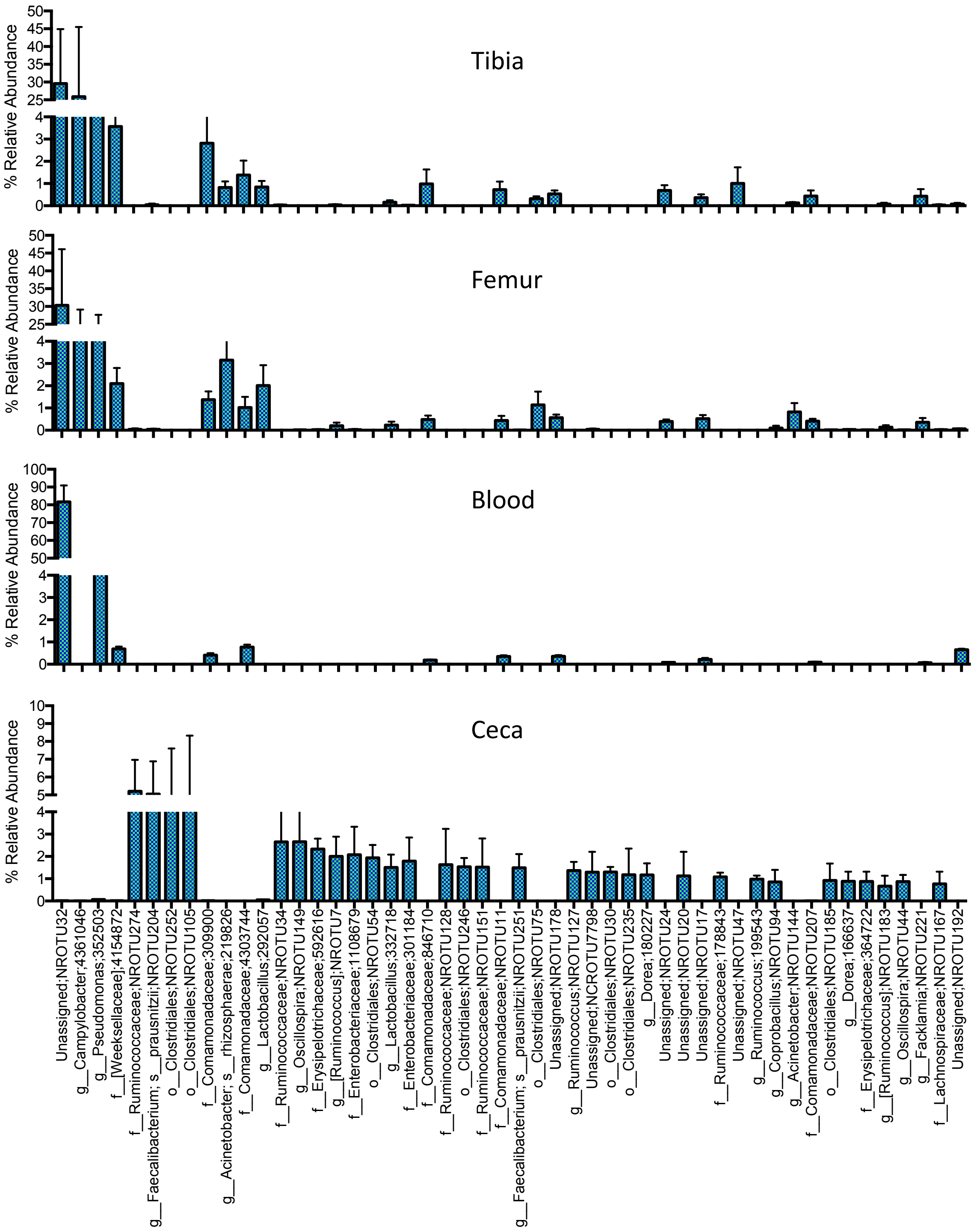

Supplemental Figure 4. Relative abundance of top 50 OTUs taken from all the sites combined on day 17. Bar indicate mean ± S.E. The highest taxonomic rank is shown followed by OTU number. O__: order, f__: family, g__: genus, s__: species, and NR-new reference.

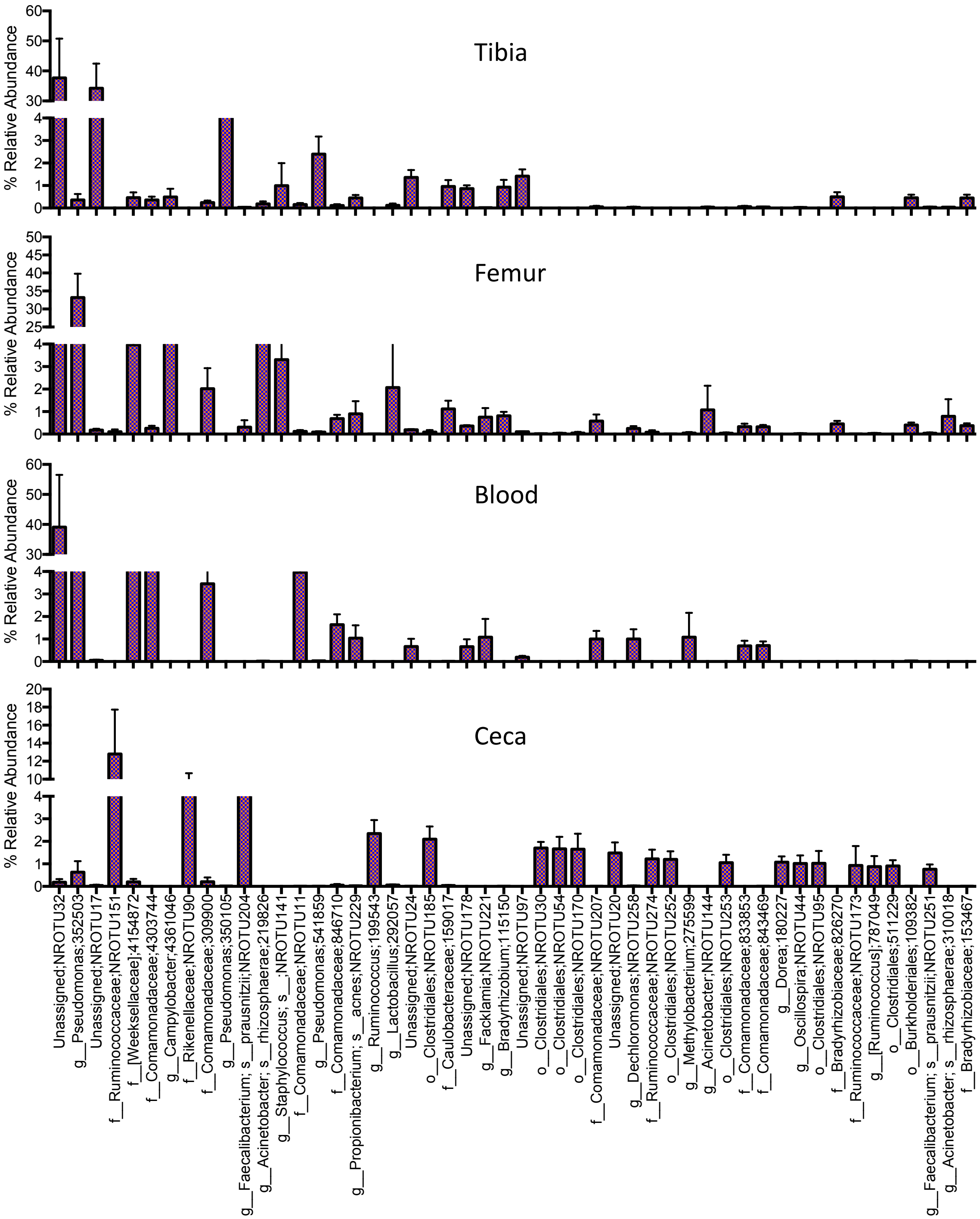

Supplemental Figure 5. Relative abundance of top 50 OTUs taken from all the sites combined on day 56. Bar indicate mean ± S.E. The highest taxonomic rank is shown followed by OTU number. O__: order, f__: family, g__: genus, s__: species, and NR- new reference.

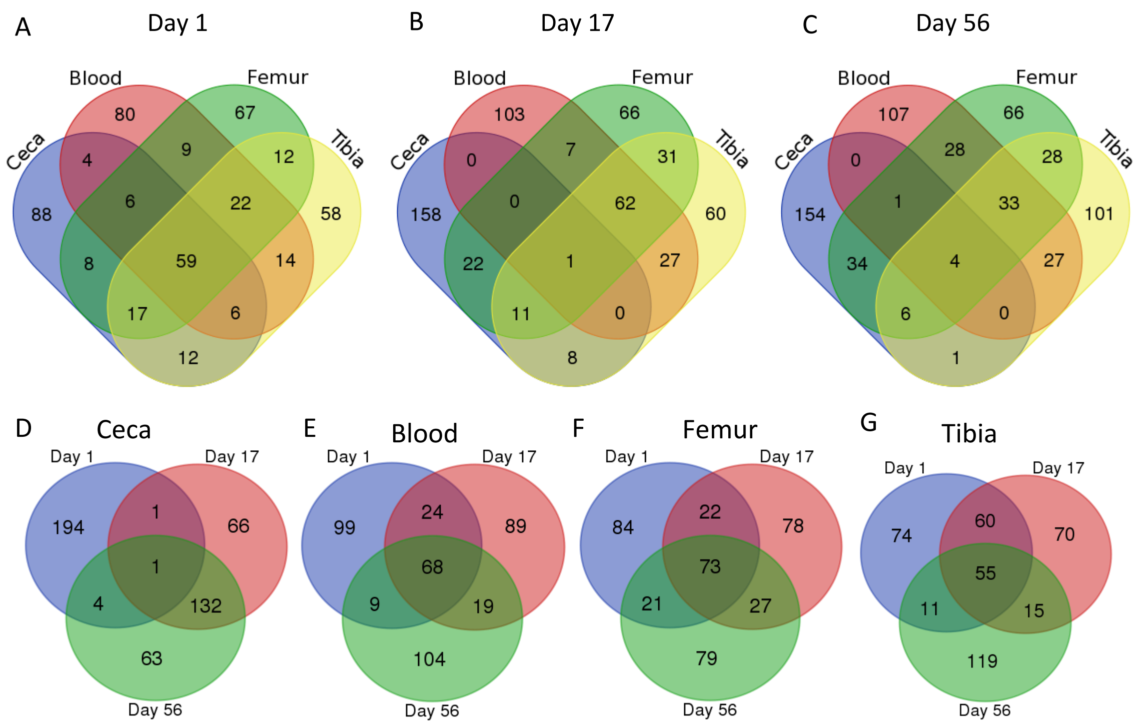

Supplemental Figure 6. Number of OTUs shared across different ages and sites of chickens. Venn diagram was constructed with top 200 abundant OTUs in every twelve specific groups (A-C) among different body sites across different ages, and (D-G) among different ages across different body sites.

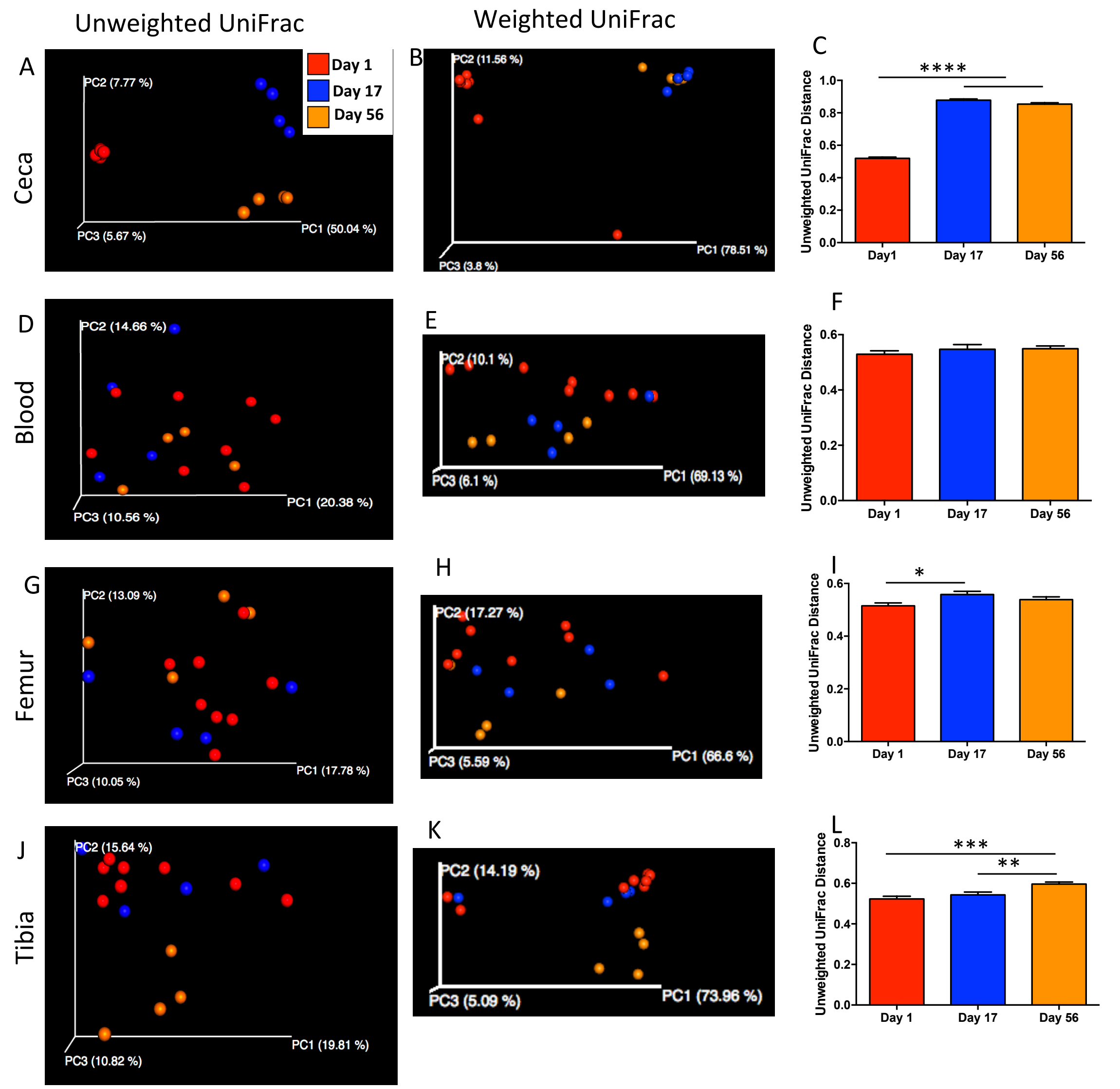

Supplemental Figure 7. Beta diversity of chicken sites across ages. Principal coordinate analysis (PCoA) plots show clustering of samples using unweighted (A, D, G, and J) and weighted (B,E,H, and K) UniFrac distance at even subsampling depth of 600 sequences/sample. Unweighted UniFrac distance of C) ceca at day 1 to ceca at day 1, day 17 and day 56; F) Blood at day 1 to blood at day 1, day 17, and day 56; I) Femur at day 1 to femur at day 1, day 17, and day 56 and L) Tibia at day 1 to tibia at day 1, day 17, and day 56. Data are mean ± standard error (S.E.). Samples were analyzed by ANOVA and Tukey's multiple-comparison test. * $P < 0.05$, ** $P < 0.01$, *** $P < 0.001$, and **** $P < 0.0001$.

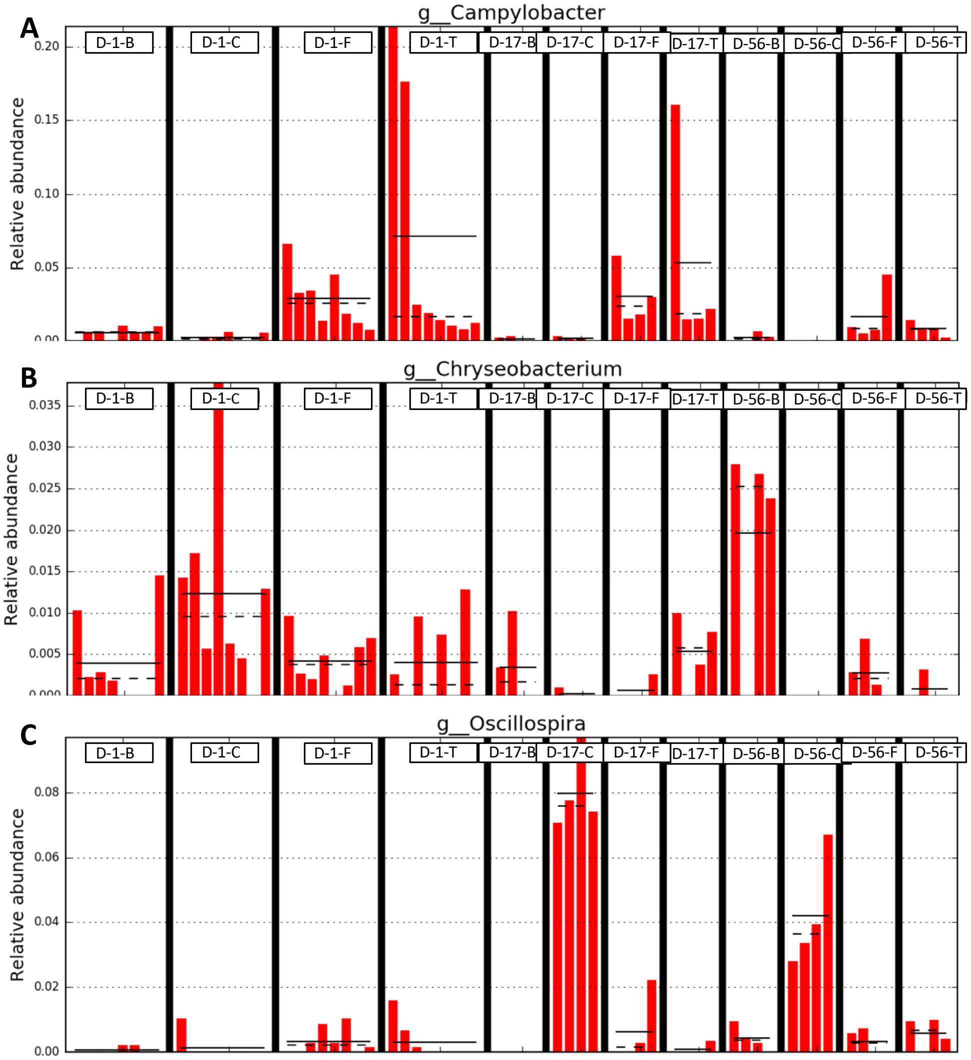

Supplemental Figure 8. Select differentially abundant features from Fig 7A. Y-axis: Relative abundance in the scale of 1. The solid horizontal line indicates the mean and dotted horizontal line indicates the median. g__: genus.

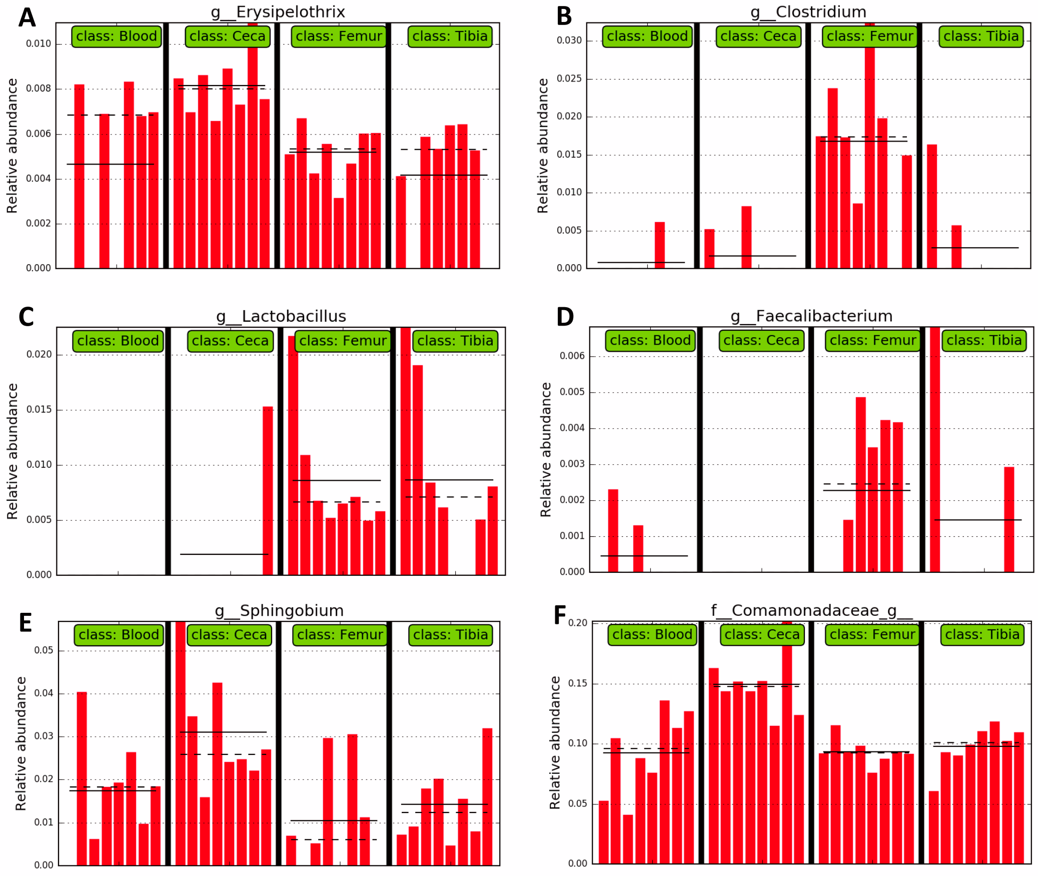

Supplemental Figure 9. Select differentially abundant features from Fig 7B. Y-axis: Relative abundance in the scale of 1. The solid horizontal line indicates the mean and dotted horizontal line indicates the median. g__: genus.

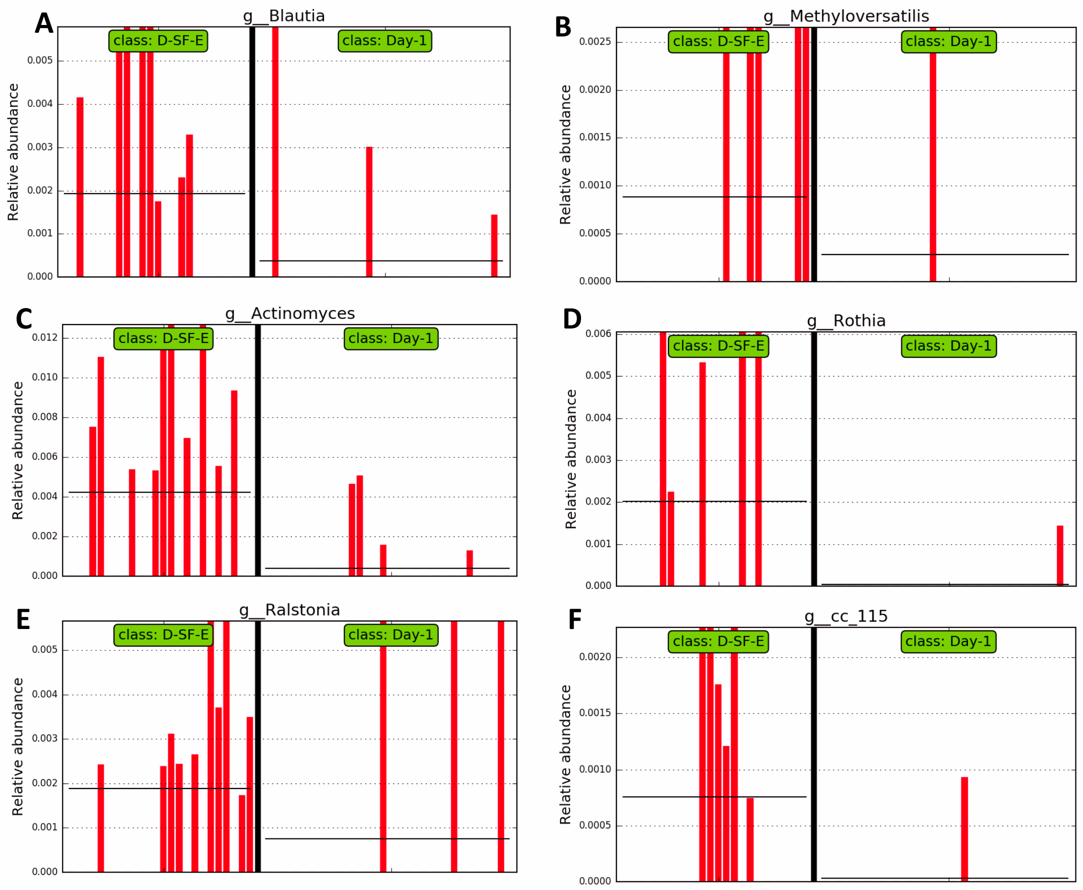

Supplemental Figure 10. Select differentially abundant features from Fig 7C. Y-axis: Relative abundance in the scale of 1. Solid and dotted horizontal line indicates mean and median respectively. g__: genus. D-SG-E: Day 17 and day 56 extraintestinal sites.

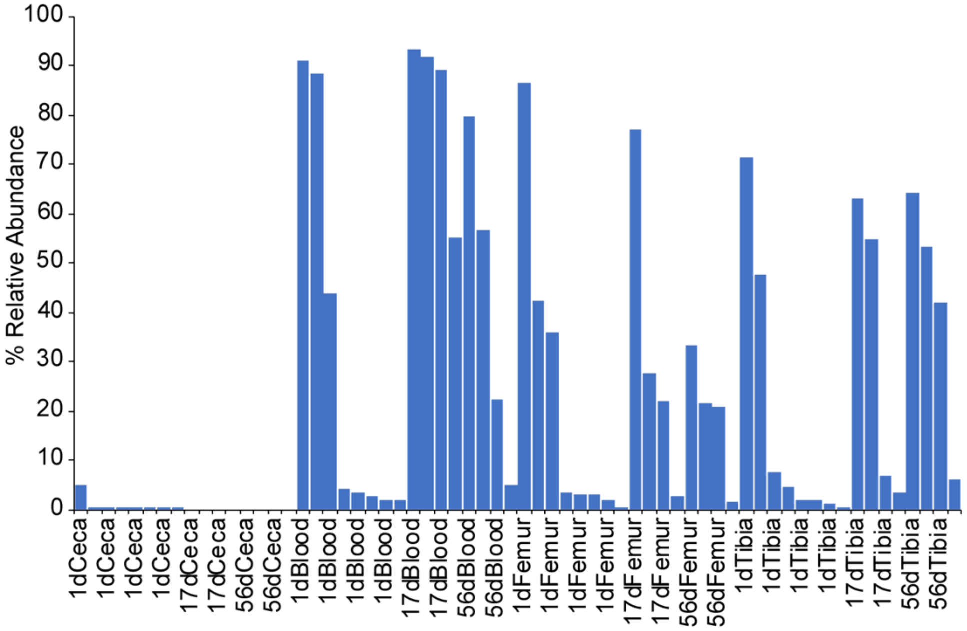

Supplemental Figure 11: Relative abundance of unassigned OTUs that significantly matched to reference genome of chicken. 1d - 1 day ; 17d – 17 day; and 56d – 56 day old chickens.

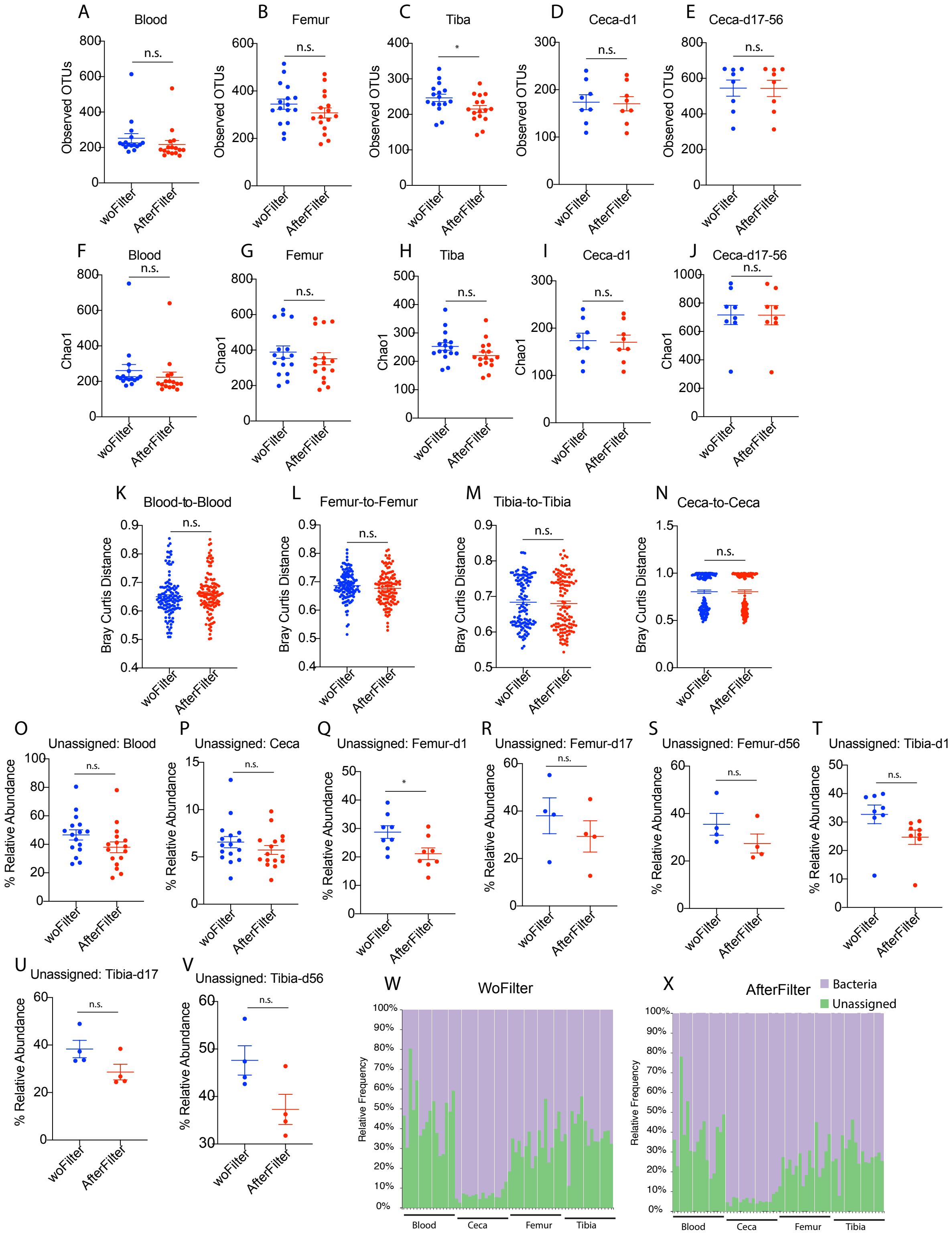

Supplementary Figure 12: The effect of filtering out unassigned OTUs that considerably matched to chicken genome was very minimal on diversity indices and taxonomic assignment. Alpha diversity measured with observed OTUs (A-E) and

chao1 index (F-J). Beta diversity measured using Bray Curtis distance (K-N). Relative abundance of unassigned OTUs (O-V). Bar graph at level 1 (W and X). Data are mean mean ± S.E. WoFilter: Without filtering the unassigned OTUs with significant match to chicken genome. AfterFilter: Filtering out the unassigned OTUs with significant match to chicken genome. Statistical analysis were performed using unpaired T test. *: $P < 0.05$. n.s.: non-significant.

Supplemental Table 1: Summary of sequencing and alpha diversity metrics of chickens in this study. * CSS normalized.

#SampleID	Body Site	Health Status	Age (Day)	# Sequences	# Sequences*	observed_otus*	chao1*	PD_whole_tree*
1d1B	Blood	Healthy	1	32850	1326.7221	295	295	4.6425
1d1C	Ceca	Healthy	1	32636	1036.5637	191	191	7.4042
1d1F	Femur	Healthy	1	33496	1193.2196	262	262	7.72946
1d1T	Tibia	Healthy	1	99082	916.6704	261	261	9.75638
1d2B	Blood	Healthy	1	147499	1109.0306	218	218	5.12765
1d2C	Ceca	Healthy	1	21539	1015.3255	183	183	7.08919
1d2F	Femur	Healthy	1	74194	1011.7397	198	198	5.68705
1d2T	Tibia	Healthy	1	68722	1064.2442	235	235	7.82652
1d3B	Blood	Healthy	1	12137	1703.2763	614	751.6571429	5.68502
1d3C	Ceca	Healthy	1	26287	910.3737	157	157	6.3847
1d3F	Femur	Healthy	1	25479	1473.531	318	318	9.43148
1d3T	Tibia	Healthy	1	34988	1538.4797	328	328	8.10804
1d4B	Blood	Healthy	1	55139	1626.1908	346	346	6.72918
1d4C	Ceca	Healthy	1	19534	863.8814	158	158	6.84131
1d4F	Femur	Healthy	1	42227	1867.5	417	588.1956522	11.77221
1d4T	Tibia	Healthy	1	29924	1473.0905	302	302	7.78248
1d5B	Blood	Healthy	1	39840	1113.6123	214	214	4.86511
1d5C	Ceca	Healthy	1	22040	569.6048	109	109	6.63153
1d5F	Femur	Healthy	1	67017	1850.9228	433	588.2777778	12.62193
1d5T	Tibia	Healthy	1	28182	1109.7567	229	229	6.4459
1d6B	Blood	Healthy	1	126320	1070.1193	211	211	6.78426
1d6C	Ceca	Healthy	1	26615	1181.8023	222	222	8.33721
1d6F	Femur	Healthy	1	58346	1701.5968	369	369	10.06073
1d6T	Tibia	Healthy	1	58197	1227.0105	249	249	6.45885

S1 Table: Continued.

#SampleID	Body Site	Health Status	Age (Day)	# Sequences	# Sequences*	observed_otus*	chao1*	PD_whole_tree*
1d7B	Blood	Healthy	1	24356	1170.3838	229	229	5.93638
1d7C	Ceca	Healthy	1	11319	763.0743	129	129	5.39506
1d7F	Femur	Healthy	1	51370	1586.924	327	327	8.69593
1d7T	Tibia	Healthy	1	34010	1296.3857	288	381.8571429	5.95788
1d8B	Blood	Healthy	1	14637	1113.2495	224	224	6.12542
1d8C	Ceca	Healthy	1	32085	1298.534	240	240	8.81846
1d8F	Femur	Healthy	1	52441	1511.3771	319	319	7.35201
1d8T	Tibia	Healthy	1	30472	1165.9507	228	228	6.67531
17d1B	Blood	Healthy	17	110768	1061.7555	207	207	4.54725
17d1C	Ceca	Healthy	17	24302	1954.8999	472	694.2830189	13.09854
17d1F	Femur	Healthy	17	28929	1132.838	221	221	6.03304
17d1T	Tibia	Healthy	17	38645	987.2583	236	236	7.11691
17d2B	Blood	Healthy	17	116923	980.2152	203	203	4.95175
17d2C	Ceca	Healthy	17	33734	2199.2975	581	671.2	14.36348
17d2F	Femur	Healthy	17	47853	1237.1025	265	265	5.83688
17d2T	Tibia	Healthy	17	24376	1128.8134	256	256	7.50057
17d3B	Blood	Healthy	17	69075	1073.2648	216	216	5.63155
17d3C	Ceca	Healthy	17	22292	2265.2069	623	773.828125	17.53896
17d3F	Femur	Healthy	17	34714	1573.2637	342	403.9565217	8.61991
17d3T	Tibia	Healthy	17	18180	1294.6724	267	267	8.27334
17d4B	Blood	Healthy	17	76280	1060.6272	216	216	4.95184
17d4C	Ceca	Healthy	17	32022	2312.2045	652	770.0127389	16.91231
17d4F	Femur	Healthy	17	48871	1920.44	481	599.8133333	13.03314
17d4T	Tibia	Healthy	17	30547	1253.3569	272	272	7.60974

Supplementary Table 1: Continued.

#SampleID	Body Site	Health Status	Age (Day)	# Sequences	# Sequences*	observed_otus*	chao1*	PD_whole_tree*
56d1B	Blood	BCO	56	93536	1354.6944	258	258	10.30809
56d1C	Ceca	BCO	56	2757	1066.3478	317	317	12.51961
56d1F	Femur	BCO	56	61854	1688.032	354	354	9.59371
56d1T	Tibia	BCO	56	24679	1077.7423	229	229	5.69048
56d2B	Blood	BCO	56	102028	1202.5875	226	226	8.28399
56d2C	Ceca	BCO	56	2615	1251.7775	413	661.8815789	12.74169
56d2F	Femur	BCO	56	57696	2162.0196	515	627.4831461	12.30646
56d2T	Tibia	BCO	56	15954	900.2812	170	170	5.48451
56d3B	Blood	BCO	56	46556	1005.5443	176	176	6.62431
56d3C	Ceca	BCO	56	24384	2279.5502	648	906.3783784	18.29908
56d3F	Femur	BCO	56	52603	1429.8645	320	405	6.87886
56d3T	Tibia	BCO	56	14583	900.744	177	177	5.32871
56d4B	Blood	BCO	56	29780	1035.7815	185	185	4.75959
56d4C	Ceca	BCO	56	19736	2250.3696	653	938.4660194	18.35002
56d4F	Femur	BCO	56	51788	1621.9954	371	371	10.30528
56d4T	Tibia	BCO	56	8274	983.5337	216	216	9.07932

16S rRNA gene DNA sequence of top fifteen most frequent OTUs in this study from Supplemental Figure. 3B (FASTA format)

Representative fasta sequence of each OTU is shown followed by top blastn hit against Reference RNA sequences (refseq_rna) database. The bacterial nomenclature is followed by max score, total score, query coverage, E value, Identities, and accession number repectivley.

>352503 56d2F_665; g_Pseudomonas
AGGGTTTGATCCTGGCTCAGATTGAACGCTGGCGGCAGGCCTAACACATGCAAGTCG
AGCGGTAGAGAGGAGCTTGCTCCTCTTGAGAGCGGCGGACGGGTGAGTAATGCCTA
GGAATCTGCCTGGTAGTGGGGGATAACGTTCGGAAACGGACGCTAATACCGCATAC
GTCCTACGGGAGAAAGCAGGGGACCTTCGGGCCTTGCGCTATCAGATGAGCCTAGGT
CGGATTAGCTAGTTGGTGAGGTAATGGCTCACCAAGGCGACGATCCGTAACTGGTCT
GAGAGGATGATCAGTCACACTGGAACTGAGACACGGTCCAGACTCCTACGGGAGGC
AGCAGTGGGGAATATTGGACAATGGGCGAAAGCCTGATCCAGCCATGCCGCGTGTG
TGAAGAAGGTCTTCGGATTGTAAAGCACTTTAAGTTGGGAGGAAGGGCAGTAAATTA
ATACTTTGCTGTTTTGACGTTACCGACAGAATAAGCACCGGCTAACTCTGTGCCAGC
AGCCGCGGTAA

Pseudomonas panacis strain CG20106 16S ribosomal RNA gene, partial sequence
939 939 99% 0.0 99% NR_043195.1

>4154872 1d1C_548; f__[Weeksellacease]
AGGGTTTGATCCTGGCTCAGGATGAACGCTAGCGGGAGGCCTAACACATGCAAGCC
GAGCGGTATTGTTTCTTCGGAAATGAGAGAGCGGCGTACGGGTGCGGAACACGTGTG
CAACCTGCCTTTATCTGGGGGATAGCCTTTCGAAAGGAAGATTAATACTCCATAATA
TATTGAACGGCATCGTTTAATATTGAAAGCTCCGGCGGATAGAGATGGGCACGCGCA
AGATTAGCTAGTTGGTGAGGTAACGGCTCACCAAGGCGATGATCTTTAGGGGGCCTG
AGAGGGTGATCCCCCACACTGGTACTGAGACACGGACCAGACTCCTACGGGAGGCA
GCAGTGAGGAATATTGGTCAATGGGTGCAAGCCTGAACCAGCCATCCCGCGTGAAG
GACGACTGCCCTATGGGTTGTAAACTTCTTTTGTATAGGGATAAACCTACCCTCGTGA
GGGTAGCTGAAGGTACTATACGAATAAGCACCGGCTAACTCCGTGCCAGCAGCCGC
GGTAA

Cloacibacterium rupense strain NBRC 104931 16S ribosomal RNA gene, partial sequence
887 887 96% 0.0 99% NR_114274.1

>New.ReferenceOTU32 17d6B_506067
AGGGTTTGATCCTGGCTCAGGAAGATAACAGTAGCTGAGCTGGATGGAACCAGATC
ACGTATCTGGGCTGAGTCCTGGTGGTGCCTGCACCAACGCCTCGGGGAGCTCGTGGC
TGCTCGCTGTGGGAAGTGTGTGGTTCTTCCATGCACAGCCTTCCCAGCGGGGACTGA
AGACTGGGAACCAGTACATATAGTACAGGTATTTTTAGCCTAAAGATTTTCATTTCAT
TATAGAATTGGCTAGTCTTTGCTGTCTCCTCACTTTTGAACACTAGTGGGTCCTAGAC
TGCATGGCACCTTGATTTAAGTCATATACGTATCAAAAGCTAGTAACCCTGAGATCA
TTCAAGCACAAAAAGCCTTTGGTCTTGCAGGGTTGCTTCACTGCTAAAAGGAGAGGA
CAGGCTTCCACCAAACTGCCTTTGCTGGGATCACATTGCTGGCCAAGCCCTGTGCCA
GCAGCCGCGGTAA

No significant similarity found.

>309900 56d5B_643; f__Comamonadaceae
AGGGTTTGATTCTGGCTCAGATTGAACGCTGGCGGCATGCCTTACACATGCAAGTCG
AACGGTAACAGGTCTTCGGATGCTGACGAGTGGCGAACGGGTGAGTAATACATCGG
AACGTGCCCGATCGTGGGGGATAACGAAGCGAAAGCTTTGCTAATACCGCATAAGA
TCTACGGATGAAAGCAGGGGACCGCAAGGCCTTGCGCGAACGGAGCGGCCGATGGC
AGATTAGGTAGTTGGTGGGATAAAAGCTTACCAAGCCGACGATCTGTAGCTGGTCTG
AGAGGACGACCAGCCACACTGGGACTGAGACACGGCCCAGACTCCTACGGGAGGCA
GCAGTGGGGAATTTTGGACAATGGGCGAAAGCCTGATCCAGCCATGCCGCGTGCAG
GATGAAGGCCTTCGGGTTGTAAACTGCTTTTGTACGGAACGAAAAGACTCTGGTTAA
TACCTGGGGTCCATGACGGTACCGTAAGAATAAGCACCGGCTAACTACGTGCCAGCA
GCCGCGGTAA

Acidovorax temperans strain PHL 16S ribosomal RNA gene, partial sequence
924 924 99% 0.0 99% NR_028715.1

>846710 1d4B_6089; f__Comamonadaceae
AGGGTTTGATCCTGGCTCAGATTGAACGCTGGCGGCATGCCTTACACATGCAAGTCG
AACGGTAACAGGTCTTCGGATGCTGACGAGTGGCGAACGGGTGAGTAATACATCGG
AACGTGCCCGATCGTGGGGGATAACGAGGCGAAAGCTTTGCTAATACCGCATACGAT
CTACGGATGAAAGCGGGGGATCTTCGGACCTCGCGCGGACGGAGCGGCCGATGGCA
GATTAGGTAGTTGGTGGGATAAAAGCTTACCAAGCCGACGATCTGTAGCTGGTCTGA
GAGGATGATCAGCCACACTGGGACTGAGACACGGCCCAGACTCCTACGGGAGGCAG
CAGTGGGGAATTTTGGACAATGGGCGAAAGCCTGATCCAGCCATGCCGCGTGCAGG
ATGAAGGCCTTCGGGTTGTAAACTGCTTTTGTACGGAACGAAAAGCCTCTTTCTAAT
AAAGAGGGGTCATGACGGTACCGTAAGAATAAGCACCGGCTAACTACGTGCCAGCA
GCCGCGGTAA

Diaphorobacter nitroreducens strain NA10B 16S ribosomal RNA gene, complete sequence
946 946 100% 0.0 99% NR_024782.1

>New.ReferenceOTU207 1d7F_1278486; f__Comamonadaceae
AGGGTTTGATCATGGCTCAGATTGAACGCTGGCGGCATGCCTTACACATGCAAGTCG
AACGGTAACAGGTCTTCGGATGCTGACGAGTGGCGAACGGGTGAGTAATACATCGG
AACGTGCCCAGACGTGGGGGATAACGAGGCGAAAGCTTTGCTAATACCGCATACGA
TCTAAGGATGAAAGCAGGGGACCGCAAGGCCTTGCGCGTTTGGAGCGGCCGATGGC
AGATTAGGTAGTTGGTGGGATAAAAGCTTACCAAGCCGACGATCTGTAGCTGGTCTG
AGAGGACGACCAGCCACACTGGGACTGAGACACGGCCCAGACTCCTACGGGAGGCA
GCAGTGGGGAATTTTGGACAATGGGCGAAAGCCTGATCCAGCAATGCCGCGTGCAG
GATGAAGGCCTTCGGGTTGTAAACTGCTTTTGTACGGAACGAAAAGGCTCTGGCTAA
TACCTGGGGCACATGACGGTACCGTAAGAATAAGCACCGGCTAACTACGTGCCAGC
AGCCGCGGTAA

Acidovorax caeni strain R-24608 16S ribosomal RNA gene, complete sequence
941 941 100% 0.0 99% NR_042427.1

>4303744 56d4B_3282;f__Comamonadaceae
AGGGTTTGATCATGGCTCAGATTGAACGCTGGCGGCATGCCTTACACATGCAAGTCG
AACGGTAACGGGTCCTTCGGGATGCCGACGAGTGGCGAACGGGTGAGTAATATATC
GGAACGTGCCCAGTAGTGGGGGATAACTGCTCGAAAGAGCAGCTAATACCGCATAC
GACCTGAGGGTGAAAGGGGGGATCGCAAGACCTCTCGCTATTGGAGCGGCCGATA
TCAGATTAGCTAGTTGGTGGGGTAAAGGCCTACCAAGGCAACGATCTGTAGTTGGTC
TGAGAGGACGACCAGCCACACTGGGACTGAGACACGGCCCAGACTCCTACGGGAGG
CAGCAGTGGGGAATTTTGGACAATGGGCGCAAGCCTGATCCAGCAATGCCGCGTGC
AGGAAGAAGGCCTTCGGGTTGTAAACTGCTTTTGTCAGGGAAGAAATCTTCTGGGCT
AATACCCCGGGAGGATGACGGTACCTGAAGAATAAGCACCGGCTAACTACGTGCCA
GCAGCCGCGGTAA

Aquabacterium olei strain NHI-1 16S ribosomal RNA, partial sequence
833 833 97% 0.0 96% NR_137381.1

>New.ReferenceOTU178 56d4B_1518014
AGGGTTTGATTCTGGCTCAGCTGCTGCTTGACATTCTCAGTCTTATGCCTCCGCCTTG
TGCGAGCCTCCTCTGGCCTCTCCAGCACAATGTCATCGGTAGTCTTGGGGCTGCCCAG
CTCTGAGGACTCAGCCTTGCTCTACAGAGAGGCGGTGCAGGGTTAAGGCTCTGCATA
CCCACAGCTCAGCTGTGGCCCTGATCTCAAATCCCATTACCAGGCCAAACCTCCCCA
CCCCAGCCAGATTCCTCCTTTCTGTTCCTCACGGTCTGATACATCACCAGACTTGCAG
GCCCTGCTCCCCACCAGACCCTCAGCAACATTACCAGCATGTCCCAGAAACTGCGGC
GTCCCAGCTTGCTCGTGGGTGTCACCGGCTCCCCAGGGCTGGCCGAAGGGGCAGAGG
TGAAGTGCAGGGGTGGAGGACAGCTTTTGCCAGCCCCACCAGTGCCCATGGCCAGAC
CCTTGGTCTCCGACATGGTTCTCCTTAGACCTGCTGTGAAGACAGAGCAAACTGTTCA
CTTCAGAGCCTTCCACTGTGACATTCATGTGCCAGCAGCCGCGGTAA

Gallus gallus seizure threshold 2 homolog (mouse) (SZT2), mRNA
316 554 53% 9e-83 100% NM_001277766.1

>New.ReferenceOTU221 1d6B_100458; g__Facklamia
AGGGTTTGATTCTGGCTCAGGACGAACGCTGGCGGCGTGCCTAATACATGCAAGTCG
AACGAACCGCAACTTGAACTTGTTCTCGTTAAGGTTAGTGGCGCACGGGTGAGTAAC
ACGTGGGGAACCTGCCCTTTAGTGGGGGATAACAGTCGGAAACGACTGCTAATACCG
CATAGACAATTTAACCGCCTGGTTAAGTTGGGAAAGGTGCTACGGCATCATTAAAGG
ATGGCCCCGCGGTGCATTAGCTAGTTGGTAAGGTAACGGCTTACCAAGGCGATGATG
CATAGCCGACCTGAGAGGGTGATCGGCCACATTGGGACTGAGACACGGCCCAAACT
CCTACGGGAGGCAGCAGTAGGGAATCTTCCGCAATGGACGCAAGTCTGACGGAGCA
ACGCCGCGTGTGTGAAGAAGGTTTTCGGATCGTAAAGCACTGTTGTTAGAGAAGAAC
GGCTACTAGAGGAAATGTTAGTAGATTGACGGTATCTAACAAGAAAGCCACGGCTA
ACTACGTGCCAGCAGCCGCGGTAA

Globicatella sanguinis strain 1152-78 16S ribosomal RNA gene, partial sequence
920 920 96% 0.0 99% NR_104716.1

>New.ReferenceOTU229 1d4B_261793;g__Propionibacterium acnes
AGAGTTCGATCCTGGCTCAGGACGAACGCTGGCGGCGTGCTTAACACATGCAAGTCG
AACGGAAAGGCCCTGCTTTTGTGGGGTGCTCGAGTGGCGAACGGGTGAGTAACACGT
GAGTAACCTGCCCTTGACTTTGGGATAACTTCAGGAAACTGGGGCTAATACCGGATA
GGAGCTCCTGCTGCATGGTGGGGGTTGGAAAGTTTCGGCGGTTGGGGATGGACTCGC
GGCTTATCAGCTTGTTGGTGGGGTAGTGGCTTACCAAGGCTTTGACGGGTAGCCGGC
CTGAGAGGGTGACCGGCCACATTGGGACTGAGATACGGCCCAGACTCCTACGGGAG
GCAGCAGTGGGGAATATTGCACAATGGGCGGAAGCCTGATGCAGCAACGCCGCGTG
CGGGATGACGGCCTTCGGGTTGTAAACCGCTTTCGCCTGTGACGAAGCGTGAGTGAC
GGTAATGGGTAAAGAAGCACCGGCTAACTACGTGCCAGCAGCCGCGGTAA

Propionibacterium acnes strain ATCC 6919 16S ribosomal RNA, complete sequence
922 922 99% 0.0 99% NR_040847.1

>843469 1d4T_940; f__Comamonadaceae
AGGGTTCGATCATGGCTCAGATTGAACGCTGGCGGCATGCCTTACACATGCAAGTCG
AACGGTAACAGGTCTTCGGATGCTGACGAGTGGCGAACGGGTGAGTAATACATCGG
AACGTGCCCGATCGTGGGGGATAACGAGGCGAAAGCTTTGCTAATACCGCATACGAT
CTACGGATGAAAGCGGGGGATCTTCGGACCTCGCGCGGACGGAGCGGCCGATGGCA
GATTAGGTAGTTGGTGGGATAAAAGCTTACCAAGCCGACGATCTGTAGCTGGTCTGA
GAGGATGATCAGCCACACTGGGACTGAGACACGGCCCAGACTCCTACGGGAGGCAG
CAGTGGGGAATTTTGGACAATGGGCGAAAGCCTGATCCAGCCATGCCGCGTGCAGG
ATGAAGGCCTTCGGGTTGTAAACTGCTTTTGTACGGAACGAAAAGCCTCTTTCTAAT
AAAGAGGGGTCATGACGGTACCGTAAGAATAAGCACCGGCTAACTACGTGCCAGCA
GCCGCGGTAA

Diaphorobacter nitroreducens strain NA10B 16S ribosomal RNA gene, complete sequence
935 935 100% 0.0 99% NR_024782.1

>4361046 17d4F_393; f__Comamonadaceae
AGGGTTTGATCCTGGCTCAGAGTGAACGCTGGCGGCGTGCCTAATACATGCAAGTCG
AACGATGAAGCTTTTAGCTTGCTAGAAGTGGATTAGTGGCGCACGGGTGAGTAAGGT
ATAGTTAATCTGCCCTACACAAGAGGACAACAGTTGGAAACGACTGCTAATACTCTA
TACTCCTGCTTAACACAAGTTGAGTAGGGAAAGTTTTTCGGTGTAGGATGAGACTAT
ATAGTATCAGCTAGTTGGTAAGGTAATGGCTTACCAAGGCTATGACGCTTAACTGGT
CTGAGAGGATGATCAGTCACACTGGAACTGAGACACGGTCCAGACTCCTACGGGAG
GCAGCAGTAGGGAATATTGCGCAATGGGGGAAACCCTGACGCAGCAACGCCGCGTG
GAGGATGACACTTTTCGGAGCGTAAACTCCTTTTCTTAGGGAAGAATTCTGACGGTA
CCTAAGGAATAAGCACCGGCTAACTCCGTGCCAGCAGCCGCGGTAA

Campylobacter subantarcticus strain LMG 24377 16S ribosomal RNA gene, partial sequence
902 902 100% 0.0 99% NR_115081.1

>833853 17d5F_1951; f__Comamonadaceae
AGGGTTTGATCCTGGCTCAGATTGAACGCTGGCGGCATGCCTTACACATGCAAGTCG
AACGGTAACAGGTCTTCGGATGCTGACGAGTGGCGAACGGGTGAGTAATACATCGG
AACGTGCCCGATCGTGGGGGATAACGAAGCGAAAGCTTTGCTAATACCGCATAAGA
TCTACGGATGAAAGCAGGGGACCGCAAGGCCTTGCGCGAACGGAGCGGCCGATGGC
AGATTAGGTAGTTGGTGGGATAAAAGCTTACCAAGCCGACGATCTGTAGCTGGTCTG
AGAGGACGACCAGCCACACTGGGACTGAGACACGGCCCAGACTCCTACGGGAGGCA
GCAGTGGGGAATTTTGGACAATGGGCGAAAGCCTGATCCAGCCATGCCGCGTGCAG
GATGAAGGCCTTCGGGTTGTAAACTGCTTTTGTACGGAACGAAAAGACTCTGGTTAA
TACCTGGGGTCCATGACGGTACCGTAAGAATAAGCACCGGCTAACTACGTGCCAGCA
GCCGCGGTAA

Acidovorax temperans strain PHL 16S ribosomal RNA gene, partial sequence
929 929 99% 0.0 99% NR_028715.1

>New.ReferenceOTU11 1d3T_74037; f__Comamonadaceae
AGGGTTCGATTCTGGCTCAGATTGAACGCTGGCGGCATGCCTTACACATGCAAGTCG
AACGGTAACGGGTCCTTCGGGATGCCGACGAGTGGCGAACGGGTGAGTAATATATC
GGAACGTGCCCAGTAGTGGGGGATAACTGCTCGAAAGAGCAGCTAATACCGCATAC
GACCTGAGGGTGAAAGGGGGGGATCGCAAGACCTCTCGCTATTGGAGCGGCCGATA
TCAGATTAGCTAGTTGGTGGGGTAAAGGCCTACCAAGGCAACGATCTGTAGTTGGTC
TGAGAGGACGACCAGCCACACTGGGACTGAGACACGGCCCAGACTCCTACGGGAGG
CAGCAGTGGGGAATTTTGGACAATGGGCGCAAGCCTGATCCAGCAATGCCGCGTGC
AGGAAGAAGGCCTTCGGGTTGTAAACTGCTTTTGTCAGGGAAGAAATCTTCTGGGCT
AATACCCCGGGAGGATGACGGTACCTGAAGAATAAGCACCGGCTAACTACGTGCCA
GCAGCCGCGGTAA

Aquabacterium olei strain NHI-1 16S ribosomal RNA, partial sequence
833 833 97% 0.0 96% NR_137381.1

>New.ReferenceOTU258 17d2B_1402194; g__Dechloromonas
AGGGTTTGATTATGGCTCAGATTGAACGCTGGCGGCATGCCTTACACATGCAAGTCG
AACGGCAGCACGGACTTCGGTCTGGTGGCGAGTGGCGAACGGGTGAGTAATGTATC
GGAACGTACCTTTCAGTGGGGGATAACGTAGCGAAAGTTACGCTAATACCGCATATT
CTGTGAGCAGGAAAGCAGGGGATCGCAAGACCTTGCGCTGATTGAGCGGCCGATAT
CAGATTAGCTAGTTGGTGGGGTAAAGGCCTACCAAGGCTACGATCTGTAGCGGGTCT
GAGAGGATGATCCGCCACACTGGAACTGAGACACGGTCCAGACTCCTACGGGAGGC
AGCAGTGGGGAATTTTGGACAATGGGGGCAACCCTGATCCAGCCATGCCGCGTGAGT
GAAGAAGGCCTTCGGGTTGTAAAGCTCTTTCGGCCGGGAAGAAATCGCATCAGCTAA
TACCTGGTGTGGATGACGGTACCGGAATAAGAAGCACCGGCTAACTACGTGCCAGC
AGCCGCGGTAA

Dechloromonas agitata strain CKB 16S ribosomal RNA gene, partial sequence
915 915 95% 0.0 99% NR_024884.1
